# Spatiotemporal Distribution of HIV Self-testing Kits Purchased on the Web and Implications for HIV Prevention in China: Population-Based Study

**DOI:** 10.2196/35272

**Published:** 2022-10-04

**Authors:** Ganfeng Luo, Lingyun Su, Anping Feng, Yi-Fan Lin, Yiguo Zhou, Tanwei Yuan, Yuqing Hu, Song Fan, Yong Lu, Yingsi Lai, Qian Shi, Jun Li, Mengjie Han, Huachun Zou

**Affiliations:** 1 School of Public Health (Shenzhen) Sun Yat-sen University Shenzhen China; 2 US-China Health Summit Beijing China; 3 School of Public Health Southwest Medical University Luzhou China; 4 School of Public Health, the Key Laboratory of Environmental Pollution Monitoring and Disease Control, Ministry of Education, Guizhou Medical University Guiyang China; 5 School of Public Health Sun Yat-sen University Guangzhou China; 6 School of Geography and Planning Sun Yat-sen University Guangzhou China; 7 School of Computer Science China University of Geosciences Wuhan China; 8 National Center for AIDS/STD Control and Prevention Chinese Center for Disease Control and Prevention Beijing China

**Keywords:** HIV self-testing, spatiotemporal patterns, China, e-commerce platform

## Abstract

**Background:**

HIV self-testing (HIVST) holds great promise for expanding HIV testing. Nonetheless, large-scale data on HIVST behavior are scant. Millions of HIVST kits are sold through e-commerce platforms each year.

**Objective:**

This study aims to analyze the spatiotemporal distribution of the HIVST kit–purchasing population (HIVSTKPP) in China.

**Methods:**

Deidentified transaction data were retrieved from a leading e-commerce platform in China. A joinpoint regression model was used to examine annual trends of the HIVSTKPP rates by calculating average annual percentage change. Bayesian spatiotemporal analysis was performed to locate hot spots with HIVSTKPP rates. Spatial autocorrelation analysis and space-time cluster analysis were conducted to identify clusters of HIVSTKPP. High-high clusters of HIVSTKPP can be identified by spatial autocorrelation analysis, and high-high clusters indicate that a region and its surrounding region jointly had a higher-than-average HIVSTKPP rate. Spatial regression analysis was used to elucidate the association between the number of HIV testing facilities, urbanization ratio (the proportion of urban population in the total population), and gross domestic product per capita and the HIVSTKPP.

**Results:**

Between January 1, 2016, and December 31, 2019, a total of 2.18 million anonymous persons in China placed 4.15 million orders and purchased 4.51 million HIVST kits on the web. In each of these 4 years, the observed monthly size of the HIVSTKPP peaked in December, the month of World AIDS Day. HIVSTKPP rates per 100,000 population significantly increased from 20.62 in 2016 to 64.82 in 2019 (average annual percentage change=48.2%; *P*<.001). Hot spots were mainly located in municipalities, provincial capitals, and large cities, whereas high-high clusters and high-demand clusters were predominantly detected in cities along the southeast coast. We found positive correlations between a region’s number of HIV testing facilities, urbanization ratio, and gross domestic product per capita and the HIVSTKPP.

**Conclusions:**

Our study identified key areas with larger demand for HIVST kits for public health policy makers to reallocate resources and optimize the HIV care continuum. Further research combining spatiotemporal patterns of HIVST with HIV surveillance data is urgently needed to identify potential gaps in current HIV-monitoring practices.

## Introduction

### Background

HIV is a growing public health challenge in China, with the annual rate of persons with newly diagnosed HIV rising from 3.66 per 100,000 in 2007 to 9.35 per 100,000 in 2020 [1,2]. At the end of 2020, there were 1.053 million people living with HIV and 351,000 cumulative reported HIV-related deaths in China [2]. Although HIV testing services are widely available in China [3,4], only an estimated 68.9% of the people living with HIV were aware of their serostatus [5], which is far below the Joint United Nations Program on HIV and AIDS target of 95% of the people living with HIV knowing their HIV status [6]. Many high-risk groups, including men who have sex with men (MSM), sex workers, and people who use drugs, are reluctant to seek venue-based HIV testing services because of concerns about confidentiality, stigma, discrimination, and inconvenience [7]. Determining how to improve the HIV detection rate and promote the identification of people living with HIV has always been the focus and difficulty of AIDS epidemic prevention and control efforts.

HIV self-testing (HIVST) holds great promise for expanding HIV testing [8]. China’s 13th Five-Year Plan for HIV Prevention and Control, a national policy framework for HIV response adopted in 2017, encouraged innovative strategies to expand HIV testing, including distribution of HIVST kits through web-based platforms [9]. E-commerce platforms such as Amazon and eBay as well as Taobao and Jingdong in China are central to how people shop and purchase goods, including HIVST kits [10]. These platforms reach billions of registered users across high-, middle-, and low-income countries [10]. HIVST kits are easily available through e-commerce platforms in China [11], with >1 million kits sold on the web in 2018 [12]. Web-based purchasing of HIVST kits is common among some populations at high risk of HIV acquisition. One-third of MSM in China have purchased HIVST kits on the web [7]. In addition, a recent study showed that of 591 individuals who ever purchased HIVST kits on the web in China, 64.7% (220/340) of the heterosexual respondents, 69.9% (112/161) of the homosexual respondents, and 72% (65/90) of the bisexual respondents had engaged in unprotected sex in the last 6 months [12]. In other words, the web-based HIVST kit–purchasing behavior largely indicates that recent high-risk sexual behavior is a factor in HIV acquisition.

### Spatiotemporal Analyses

In recent decades, spatiotemporal analyses have been applied to HIV surveillance and outbreak investigation among different populations [13-16]. Applying these same principles to analyze the spatiotemporal evolution of HIVST kit–purchasing patterns can provide insight into the distribution of those at potential high risk for HIV and in need of HIVST and HIV care [17]. This information can be used to identify gaps in HIV prevention and optimize allocation of resources [17]. Identifying associations between HIVST kit purchasing and macroscopic factors such as number of HIV testing facilities, urbanization ratio (the proportion of urban population in the total population), and gross domestic product (GDP) may help in the development of interventional strategies or policy responses contextualized to different settings.

No previous publication has analyzed the spatiotemporal distribution of HIVST, and the effect of expanding HIVST based on China’s 13th Five-Year Plan for HIV Prevention and Control remains unclear. In this study, we used transaction data collected from a leading e-commerce platform to analyze the spatiotemporal patterns of the HIVST kit–purchasing population (HIVSTKPP) to uncover clusters of HIVST kit purchasing and evaluate associations with macroscopic factors in China.

## Methods

### Data Collection

Records of the sales of HIVST kits between January 1, 2016, and December 31, 2019, were retrieved from a leading e-commerce platform in China [17]. To protect the consumers’ privacy, the name of the e-commerce platform is not being disclosed. The extracted variables included (1) anonymized ID details; (2) purchase date; (3) shipping province, city, and provincial-controlled county; and (4) purchase quantity. In addition, IP addresses were not included in the original data set.

The size of the resident population, urbanization ratio, and GDP per capita (CN¥ 10,000 [US $1410]) for each shipping province, city, and provincial-controlled county were drawn from the Statistical Yearbook of China [18]. The number of HIV testing facilities in each area was collected from the Chinese Center for Disease Control and Prevention (CCDC) [19]. Maps were obtained from the National Catalogue Service for Geographic Information [20].

### Data Management

All personal identifiable information was removed or deidentified. All data were maintained entirely within a sandbox environment of the e-commerce platform.

### Inclusion and Exclusion Criteria

To minimize the impact of bulk or proxy purchasing of HIVST kits, we only included those persons who had purchased ≤48 kits cumulatively over the entire study period. We only included records with shipping addresses within mainland China. Orders shipped to Hong Kong, Macao, Taiwan, and overseas were excluded.

### Definition of HIVSTKPP

In our study, HIVSTKPP refers to anonymous persons who purchased HIVST kits from a leading e-commerce platform for their own use for HIV testing in China between January 1, 2016, and December 31, 2019.

### Statistical Analysis

The rate of HIVST kit purchasing per 100,000 population was calculated by dividing the number of anonymous persons who bought the kits by the total population in an area. When calculating the HIVSTKPP rate, we only included each anonymous person’s latest recorded purchase to avoid duplication. In this study, anonymous person was defined as a unique individual by anonymized user ID of the e-commerce platform. Data were analyzed at the regional, provincial, city, and provincial-controlled–county levels.

In China, 31 provinces are aggregated into 7 regions (North China, Northeast China, East China, Central China, South China, Southwest China, and Northwest China) based on geography, climate, economy, history, and ethnicity by the Chinese government (Textbox 1).

**Figure 1:**
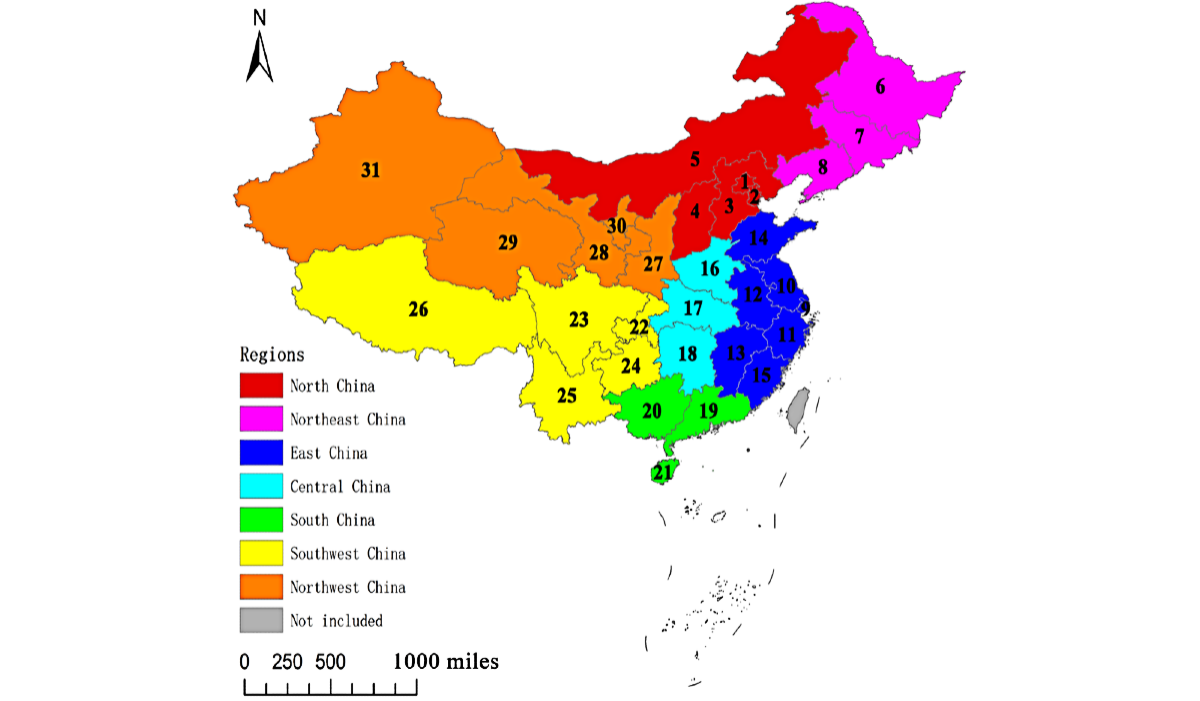
Geographical distribution of 7 regions and 31 provinces in mainland China. The 31 provinces are as follows: 1, Beijing; 2, Tianjin; 3, Hebei; 4, Shanxi; 5, Inner Mongolia; 6, Heilongjiang; 7, Jilin; 8, Liaoning; 9, Shanghai; 10, Jiangsu; 11, Zhejiang; 12, Anhui; 13, Jiangxi; 14, Shandong; 15, Fujian; 16, Henan; 17, Hubei; 18, Hunan; 19, Guangdong; 20, Guangxi; 21, Hainan; 22, Chongqing; 23, Sichuan; 24, Guizhou; 25, Yunnan; 26, Tibet; 27, Shaanxi; 28, Gansu; 29, Qinghai; 30, Ningxia; and 31, Xinjiang.

The geographical distribution of these 7 regions and 31 provinces is presented in Figure 1. In addition, 4 municipalities (Beijing, Shanghai, Tianjin, and Chongqing cities) belong to provincial administrative units. The 31 provinces and their capitals are presented in Textbox 2.

Time series analysis [21] and joinpoint regression analysis [22] were performed to examine seasonal patterns of the monthly size of the HIVSTKPP and annual trends of the HIVSTKPP rates, respectively. A spatial autocorrelation model was constructed to evaluate the spatial distribution patterns (clustered, dispersed, and random) of the HIVSTKPP [23,24]. Four types of local spatial clusters can be identified by a spatial autocorrelation model: high-high, low-low, high-low, and low-high [13]. High-high and low-low clusters indicate that a city and its surrounding region jointly had higher-than-average and lower-than-average HIVSTKPP rates, respectively. A high-low cluster represents a city with an above-average HIVSTKPP rate surrounded by cities with below-average rates, whereas a low-high cluster represents a city with a below-average HIVSTKPP rate surrounded by cities with above-average rates. Temporal-spatial clustering analysis was implemented to identify high-risk (higher demand for HIVST kits) and low-risk (lower demand for HIVST kits) clusters of HIVSTKPP over space and time simultaneously [25,26], and we set a maximum spatial cluster size of 50% of the population at risk and a maximum temporal cluster size of 50% of the study period to scan for spatial clusters with high and low rates in our study. A Bayesian spatiotemporal model was constructed to detect hot spots and cold spots of the HIVSTKPP [27]. Spatial lag model or spatial error model (SEM) and geographically weighted regression model were constructed to assess the global and local spatial correlation between the size of the HIVSTKPP and 3 factors (number of HIV testing facilities, urbanization ratio, and GDP per capita [CN¥ 10,000 {US $1410}]), respectively [13,28]. Details of the statistical analysis can be found in Multimedia Appendix 1 [1-3,6-9,17,18,29-39].

**The 7 regions and 31 provinces of mainland China.**

**Regions and provinces**

- North China
- Beijing, Tianjin, Hebei, Shanxi, and Inner Mongolia
- Northeast China
- Heilongjiang, Jilin, and Liaoning
- East China
- Shanghai, Jiangsu, Zhejiang, Anhui, Jiangxi, Shandong, and Fujian
- Central China
- Henan, Hubei, and Hunan
- South China
- Guangdong, Guangxi, and Hainan
- Southwest China
- Chongqing, Sichuan, Guizhou, Yunnan, and Tibet
- Northwest China
- Shaanxi, Gansu, Qinghai, Ningxia, and Xinjiang

**Mainland China’s 31 provincial capitals.**

**Provinces and capitals**

- Beijing: Beijing municipality
- Tianjin: Tianjin municipality
- Hebei: Shijiazhuang city
- Shanxi: Taiyuan city
- Inner Mongolia: Hohhot city
- Heilongjiang: Harbin city
- Jilin: Changchun city
- Liaoning: Shenyang city
- Shanghai: Shanghai municipality
- Jiangsu: Nanjing city
- Zhejiang: Hangzhou city
- Anhui: Hefei city
- Jiangxi: Nanchang city
- Shandong: Nanchang city
- Fujian: Fuzhou city
- Henan: Zhengzhou city
- Hubei: Wuhan city
- Hunan: Wuhan city
- Guangdong: Guangzhou city
- Guangxi: Nanning city
- Hainan: Haikou city
- Chongqing: Chongqing municipality
- Sichuan: Chengdu city
- Guizhou: Guiyang city
- Yunnan: Kunming city
- Tibet: Lhasa city
- Shaanxi: Xi’an city
- Gansu: Lanzhou city
- Qinghai: Xining city
- Ningxia: Yinchuan city
- Xinjiang: Urumqi city

### Ethics Approval

This study was conducted with the approval of the institutional review board and ethics committee of Sun Yat-sen University (SYSU-SPH2021026). We did not identify or reidentify any individual, purposefully or inadvertently, in our analysis.

## Results

### HIVST Kit–Purchasing Patterns

Between January 1, 2016, and December 31, 2019, a total of 2.18 million anonymous persons in China placed 4.15 million orders and purchased 4.51 million HIVST kits on the web. HIVST kits were delivered to 366 cities and provincial-controlled counties (Table 1). The mean number of HIVST kits sold per month was 94.01 thousand, and the mean number of HIVST kits sold per day was 3.09 thousand. Of the 2.18 million anonymous persons who purchased HIVST kits, 1.39 million (63.79%), 0.41 million (18.73%), and 0.16 million (7.39%) anonymous persons purchased HIVST kits on 1, 2, and 3 occasions between January 1, 2016, and December 31, 2019, respectively; in addition, 1.33 million (60.88%), 0.43 million (19.59%), and 0.17 million (7.66%) anonymous persons purchased 1, 2, and 3 kits, respectively (Table 2). The average annual HIVSTKPP rate between January 1, 2016, and December 31, 2019, was 39.16 (SD 19.90) per 100,000 (Table 1).

**Table 1:** The number of purchasers, purchases, and HIV self-testing kits sold as well as the HIV self-testing kit–purchasing population rate between January 1, 2016, and December 31, 2019, in mainland China.

Year	Purchasers (N=2,180,284), n (%)	Purchases (N=4,148,429), n (%)	Kits (N=4,512,353), n (%)	Population (thousand; N=5,568,220), n (%)	Rate^a^
2016	285,107 (13.08)	641,566 (15.47)	750,752 (16.64)	1,382,710 (24.83)	20.62
2017	369,140 (16.93)	832,775 (20.07)	924,661 (20.49)	1,390,080 (24.96)	26.55
2018	618,498 (28.37)	1,203,435 (29.01)	1,282,882 (28.43)	1,395,380 (25.06)	44.32
2019	907,539 (41.62)	1,470,653 (35.45)	1,554,058 (34.44)	1,400,050 (25.14)	64.82

^a^The rate of HIV self-testing kit purchasing per 100,000 population was calculated by dividing the number of purchasers by the total population in an area. The rate for the 4-year period from January 1, 2016, to December 31, 2019, was 39.16.

**Table 2:** The number of purchasers with ≥1 purchases and the number of purchasers of ≥1 HIV self-testing kits between January 1, 2016, and December 31, 2019, in mainland China.

Variable		Values (N=2,180,284), n (%)
**Purchases**		
	1	1,390,790 (63.79)
	2	408,471 (18.73)
	3	161,192 (7.39)
	4	78,898 (3.62)
	5	43,773 (2.01)
	6	27,069 (1.24)
	7	17,708 (0.81)
	8	12,174 (0.56)
	9	8804 (0.4)
	10	6469 (0.3)
**HIV self-testing kits**		
	1	1,327,298 (60.88)
	2	427,037 (19.59)
	3	167,042 (7.66)
	4	88,952 (4.08)
	5	49,154 (2.25)
	6	31,585 (1.45)
	7	20,195 (0.93)
	8	14,508 (0.67)
	9	10,689 (0.49)
	10	8364 (0.38)

### Temporal Trends in the HIVSTKPP

**Figure 2:**
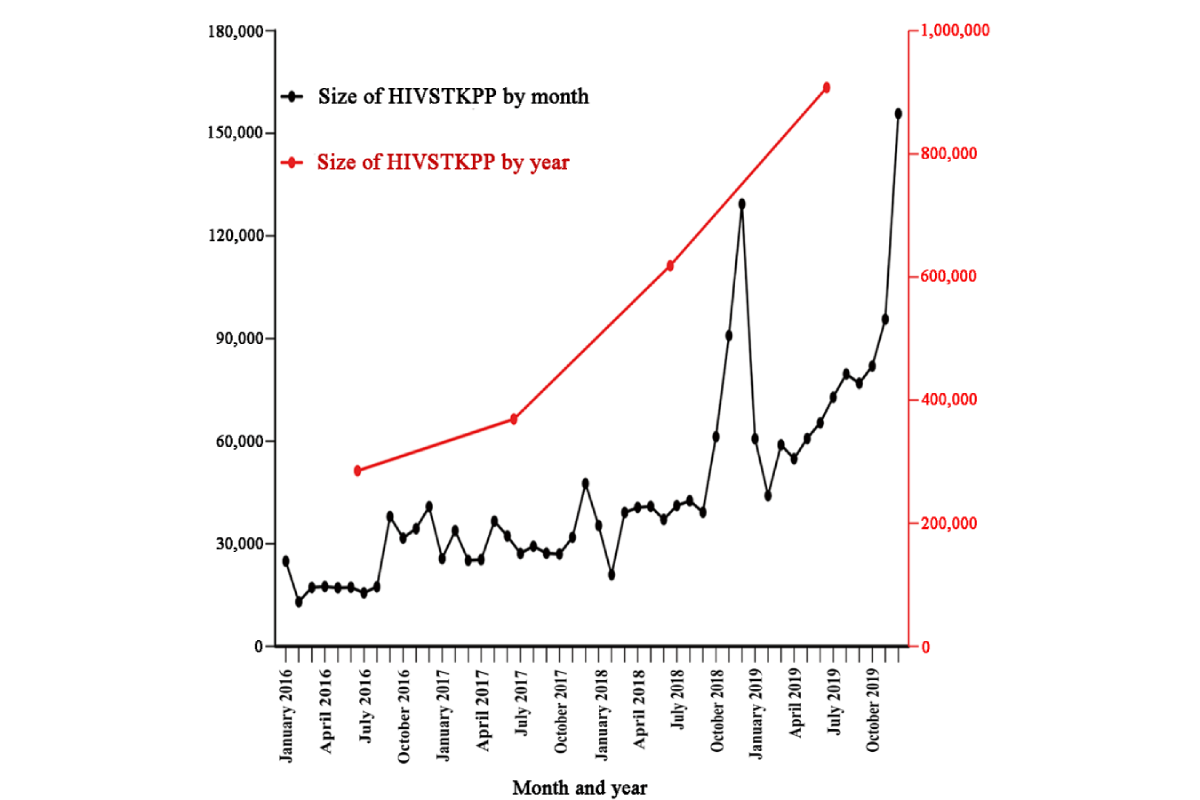
Temporal trend of the size of the HIV self-testing kit–purchasing population (HIVSTKPP) by year and month between January 1, 2016, and December 31, 2019, in mainland China.

**Figure 3:**
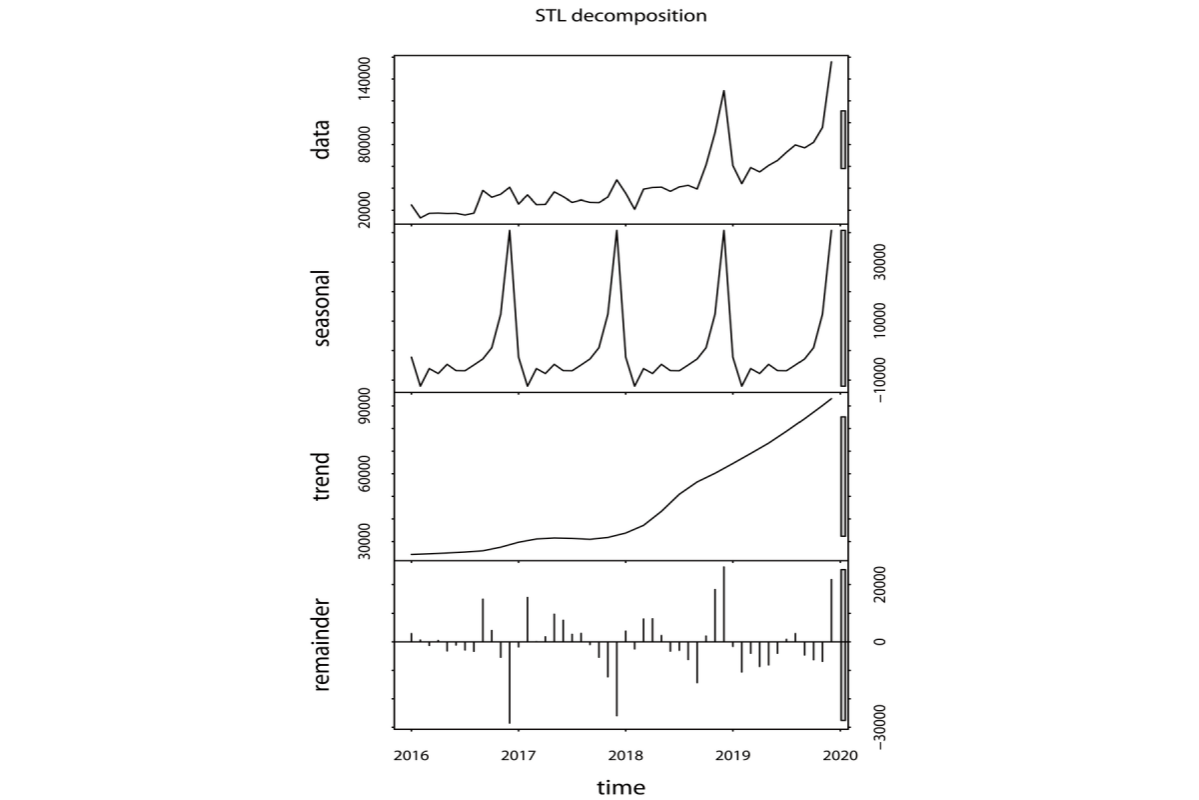
The seasonal and trend decomposition using Loess of the size of the HIV self-testing kit–purchasing population by month from January 1, 2016, to December 31, 2019, in mainland China.

**Figure 4:**
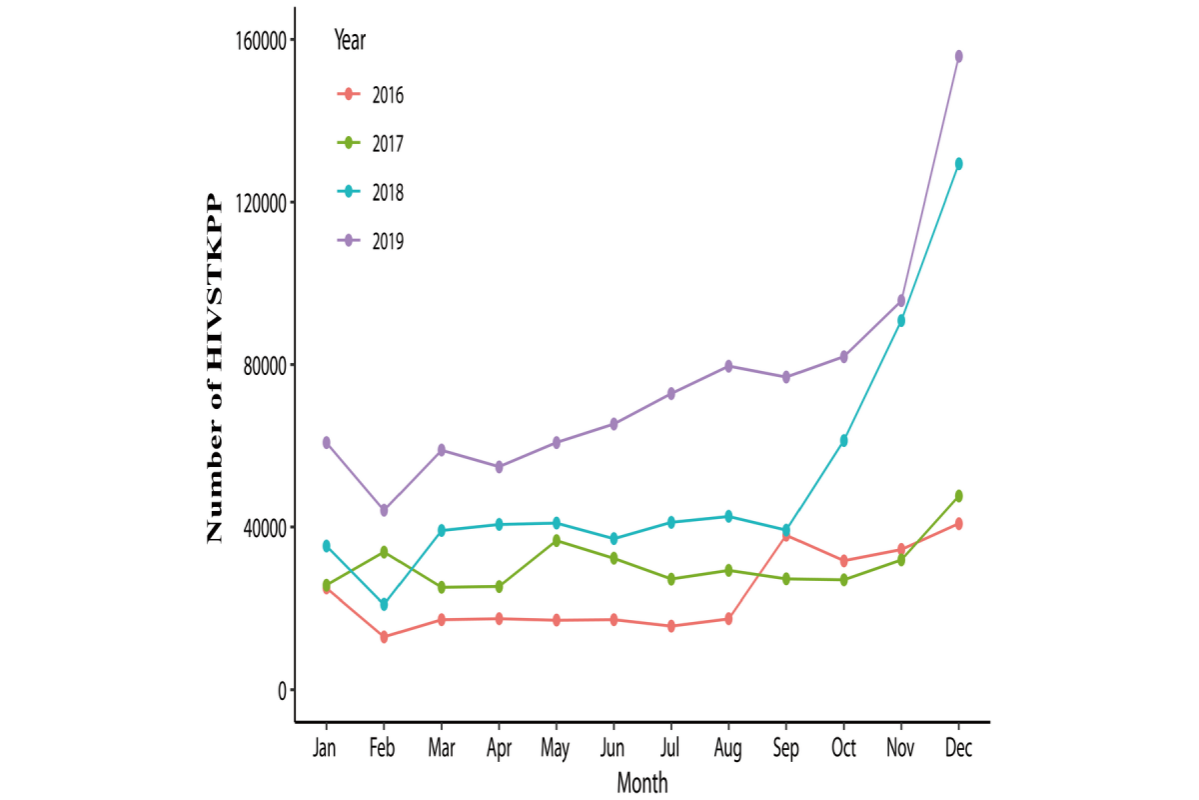
The monthly plots of the size of the HIV self-testing kit–purchasing population (HIVSTKPP) from January 1, 2016, to December 31, 2019, in mainland China.

**Figure 5:**
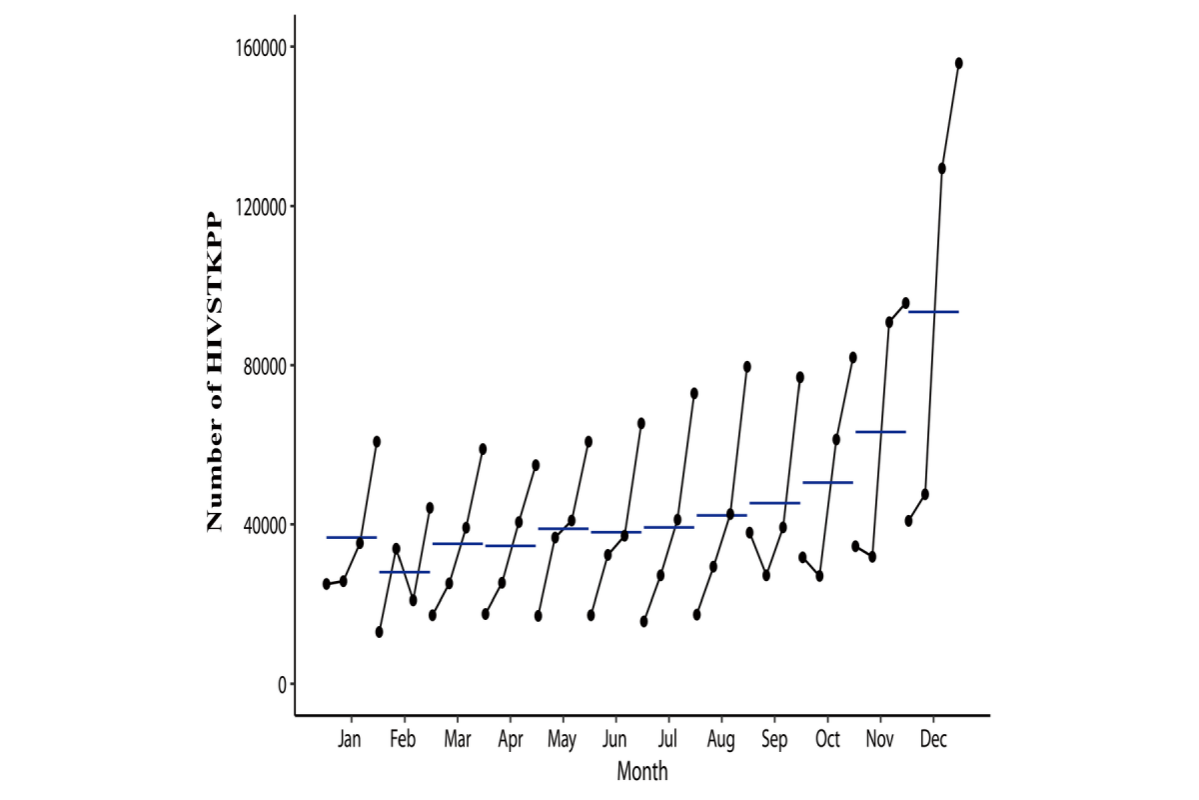
The seasonal plots of the size of the HIV self-testing kit–purchasing population (HIVSTKPP) from January 1, 2016, to December 31, 2019, in mainland China.

**Figure 6:**
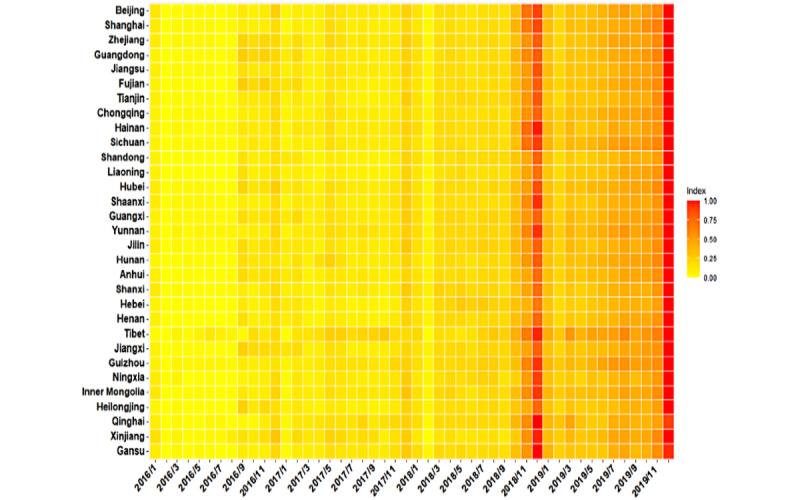
Heat map showing the size of the HIV self-testing kit–purchasing population by month after min-max normalization from January 1, 2016, to December 31, 2019, in 31 provinces in mainland China.

The observed annual HIVSTKPP markedly increased from 0.29 million anonymous persons in 2016 to 0.91 million anonymous persons in 2019 (Figure 2). The observed HIVSTKPP showed a clear periodic pattern, with 46.31% (1,009,704/2,180,284) of the anonymous persons purchasing HIVST kits between September and December (Figures 3-5). The maximum monthly number was observed in December for all provinces (Figure 5 and Figure 6).

### Temporal Trends and Spatial Distribution in the HIVSTKPP Rates per 100,000 Population

**Figure 7:**
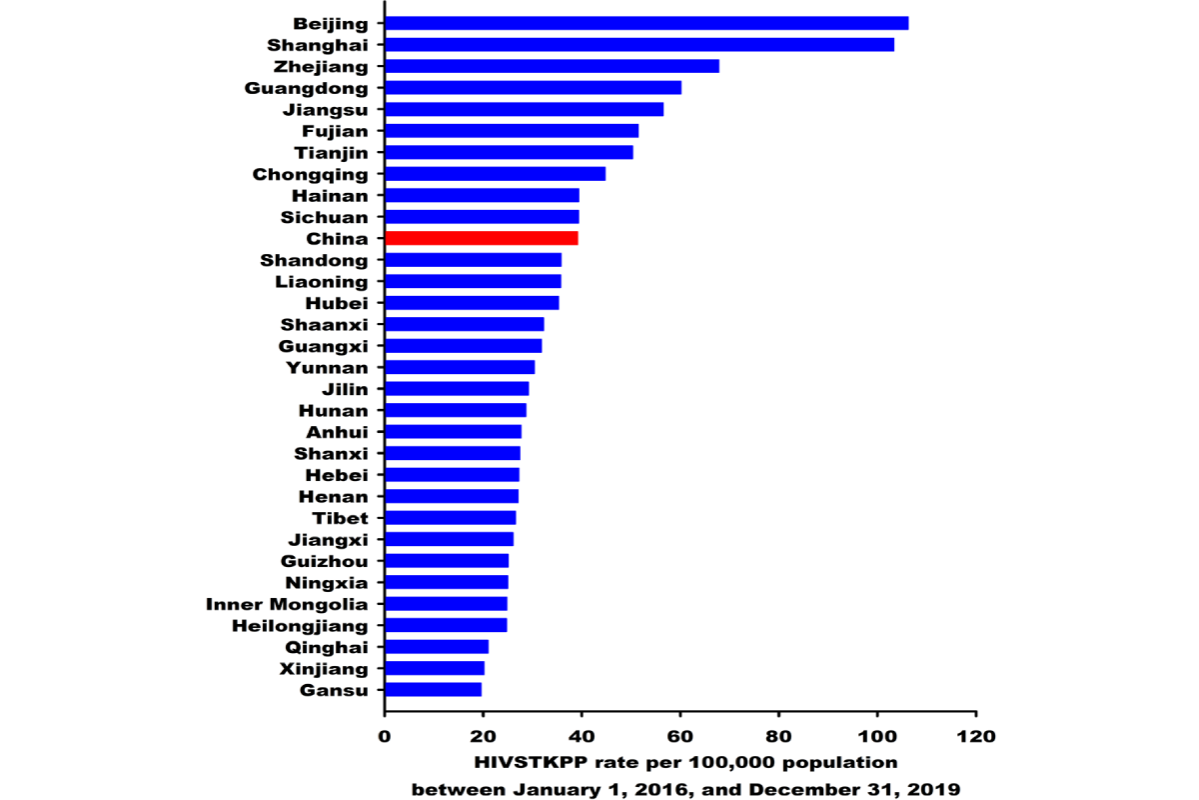
Average annual rate per 100,000 population of the HIV self-testing kit–purchasing population (HIVSTKPP) between January 1, 2016, and December 31, 2019, among the 31 provinces in mainland China.

From January 1, 2016, to December 31, 2019, the HIVSTKPP rate increased by 2.14 times (from 20.62 per 100,000 to 64.82 per 100,000, average annual percentage change [AAPC]=48.2%; *P*<.001; Table 3). Among all 7 regions in mainland China, the highest and lowest average annual HIVSTKPP rates between January 1, 2016, and December 31, 2019, were seen in South China (50.85 per 100,000) and Northwest China (24.91 per 100,000), respectively (Table 3). Among the 31 provinces, the largest sizes of HIVSTKPP were observed in Guangdong (0.27 million), Jiangsu (0.18 million), Zhejiang (0.15 million), Shandong (0.14 million), and Sichuan (0.13 million; data not shown). The highest HIVSTKPP rates were observed in Beijing (106.20), Shanghai (103.32), Zhejiang (67.79), Guangdong (60.10), and Jiangsu (56.48; Figure 7 and Table 3).

**Figure 8:**
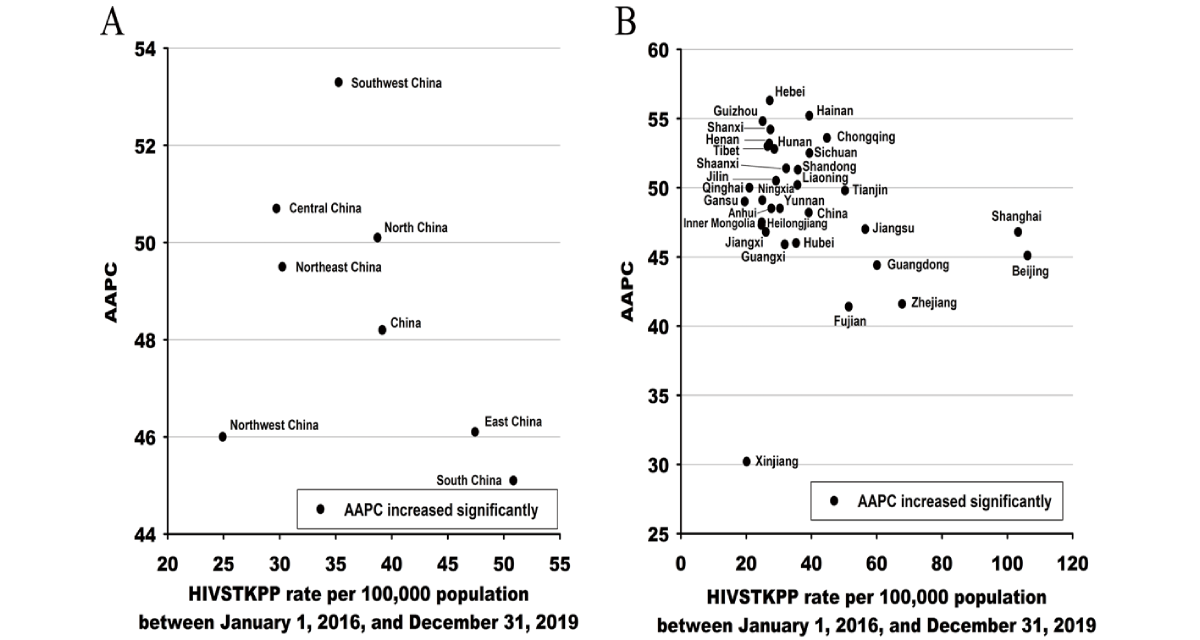
Average annual percentage change (AAPC) in the HIV self-testing kit–purchasing population (HIVSTKPP) rate per 100,000 population between January 1, 2016, and December 31, 2019, by average annual rate per 100,000 population of HIVSTKPP between January 1, 2016, and December 31, 2019, among the 7 regions (A) and 31 provinces (B) in mainland China.

**Figure 9:**
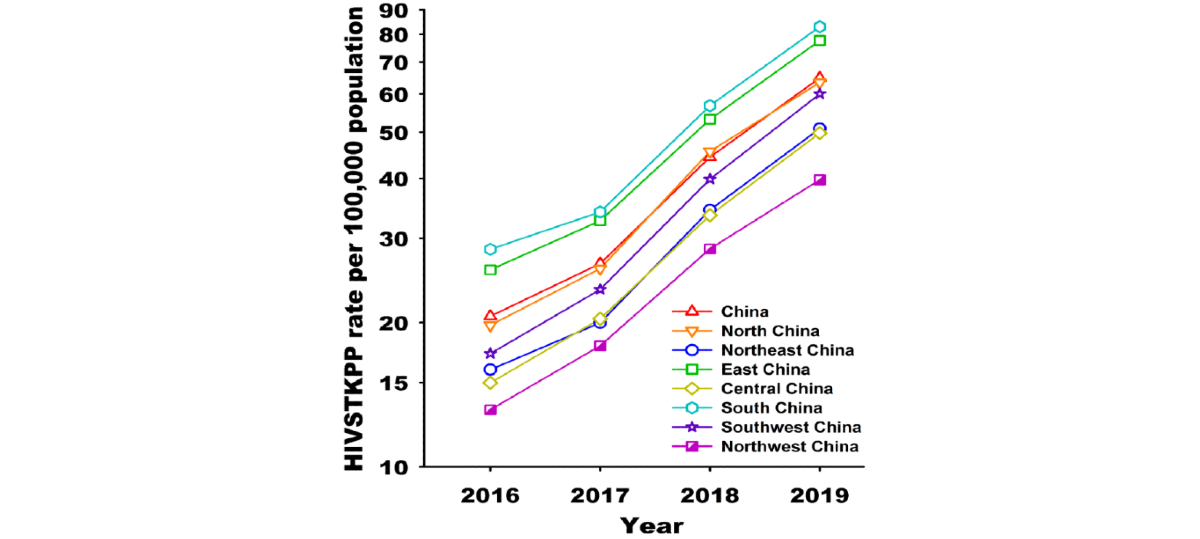
Temporal trend of the HIV self-testing kit–purchasing population (HIVSTKPP) rate per 100,000 population from January 1, 2016, to December 31, 2019, by region (refer to Textbox 1, which shows the number of regions and provinces).

**Figure 10:**
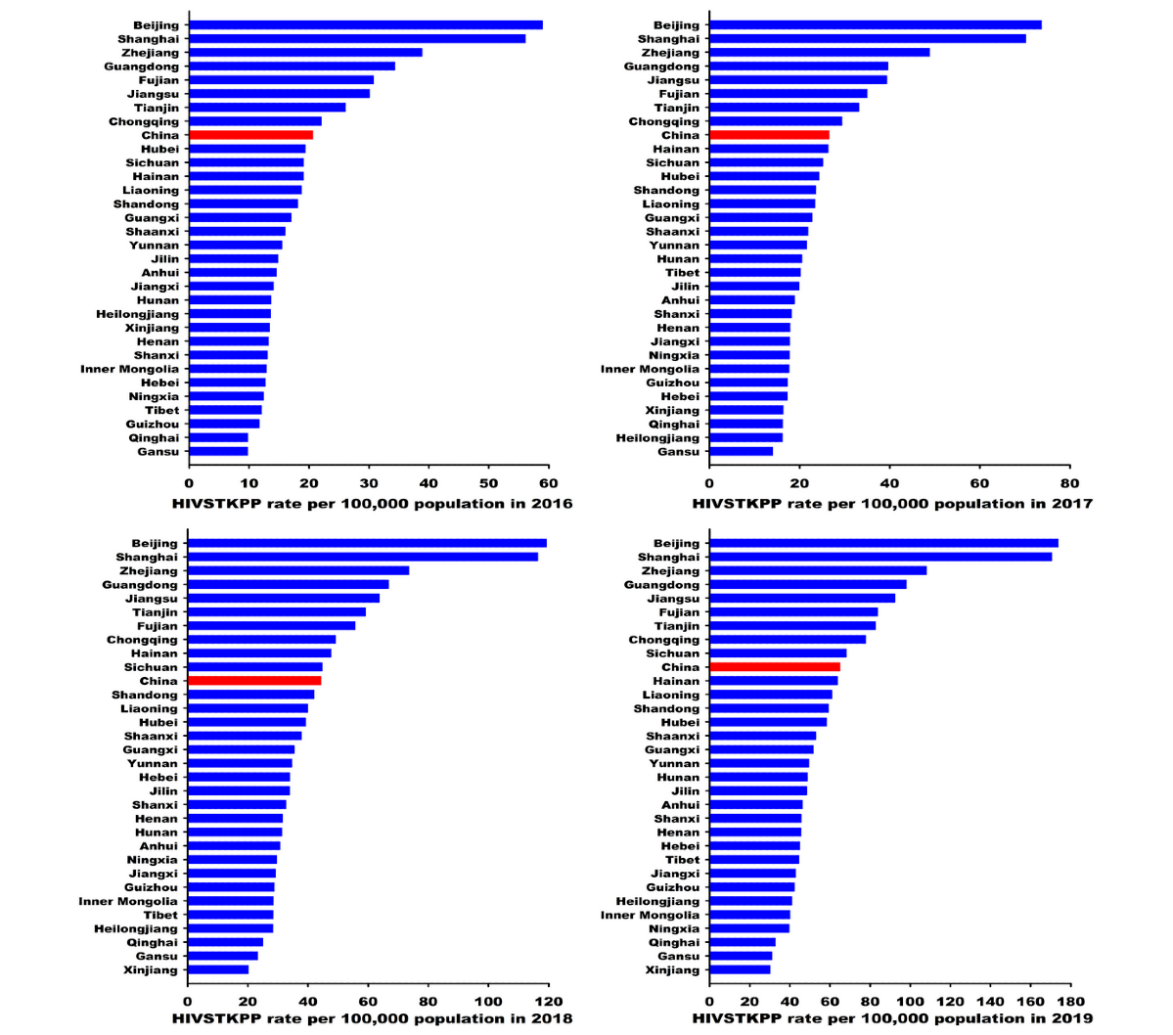
The HIV self-testing kit–purchasing population (HIVSTKPP) rate per 100,000 population from January 1, 2016, to December 31, 2019, for 31 provinces in mainland China.

**Figure 11:**
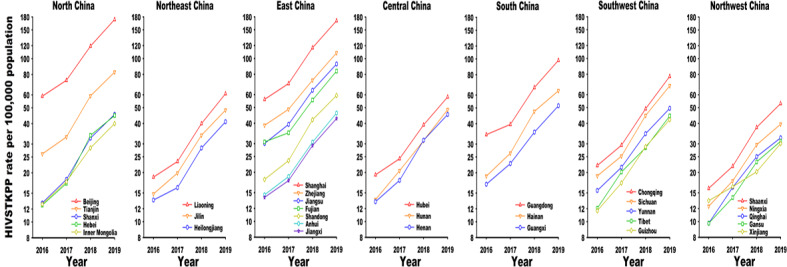
Temporal trend of the HIV self-testing kit–purchasing population (HIVSTKPP) rate per 100,000 population from January 1, 2016, to December 31, 2019, by province (there are 31 provinces in mainland China).

From January 1, 2016, to December 31, 2019, the HIVSTKPP rates significantly increased among all 7 regions and 31 provinces (all *P*<.001; Figures 8-11, and Table 3). Southwest China, with a median annual HIVSTKPP rate of 35.25 per 100,000, showed the largest increase in HIVSTKPP rates (AAPC=53.3%; *P*<.001; Figure 8A and Figure 9). Most provinces had low annual HIVSTKPP rates but exhibited large increases in HIVSTKPP rates (Figure 8B and Figure 11). Beijing and Shanghai municipalities had both high average annual HIVSTKPP rates and large increases in HIVSTKPP rates.

**Figure 12:**
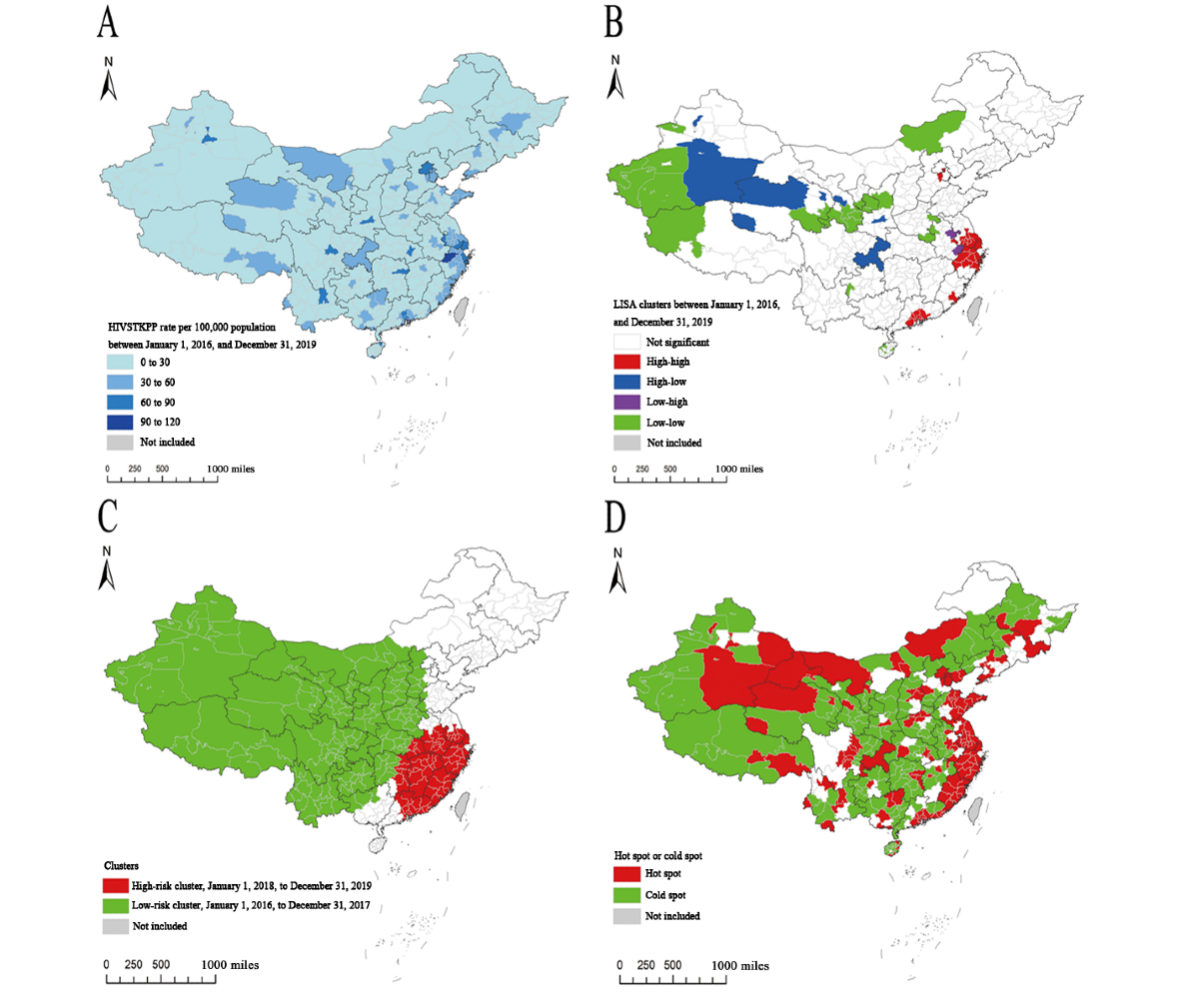
(A) Geographical distribution of the average annual rate per 100,000 population of the HIV self-testing kit–purchasing population (HIVSTKPP), (B) local clusters of the average annual rate of the HIVSTKPP identified by spatial autocorrelation analysis, (C) spatiotemporal clusters of the HIVSTKPP identified by temporal-spatial clustering analysis, and (D) hot spots and cold spots identified by the best-fitting Bayesian spatiotemporal model between January 1, 2016, and December 31, 2019, at city and provincial-controlled–county levels in mainland China. LISA: local indicators of spatial association; RR: relative risk.

**Figure 13:**
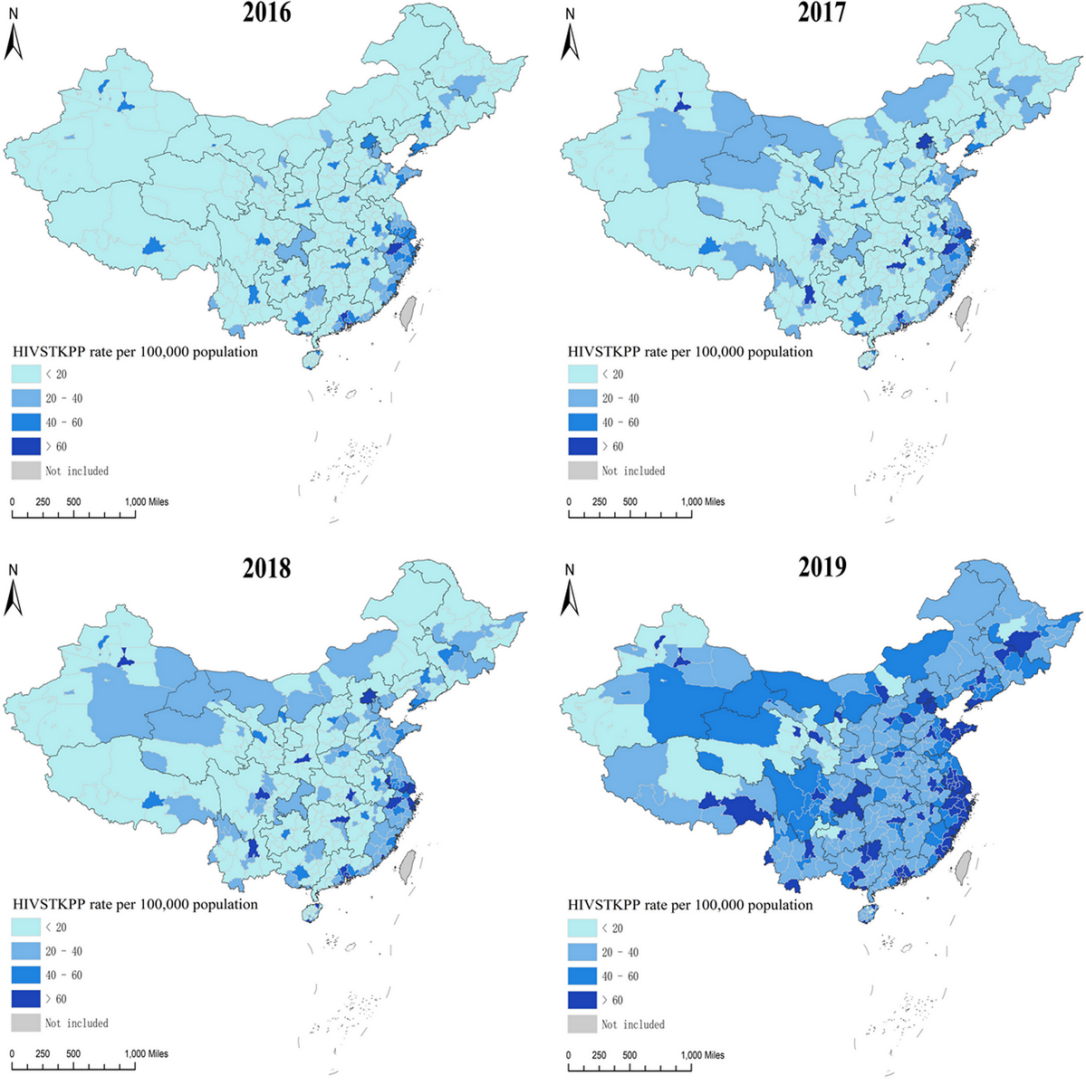
Geographical distribution of the HIV self-testing kit–purchasing population (HIVSTKPP) rate per 100,000 population from January 1, 2016, to December 31, 2019, at city and provincial-controlled–county levels in mainland China.

The spatial distributions in HIVSTKPP rates at city and provincial-controlled–county levels were distinctly heterogeneous (Figure 12A and Figure 13). The top 10 cities with the highest average annual HIVSTKPP rates are shown in Textbox 3, and those with the largest sizes of HIVSTKPP are shown in Textbox 4.

**Table 3:** Average annual percentage change (AAPC) in HIV self-testing kit–purchasing population rates per 100,000 population between January 1, 2016, and December 31, 2019, among the 7 regions and 31 provinces in mainland China.

							Average annual HIVSTKPP^a^ rates per 100,000 population											AAPC (%)^b^			*P* value
							2016 to 2019		2016		2017		2018		2019						
China							39.16		20.62		26.56		44.32		64.82		48.2			<.001	
**North China**							38.73		19.75		25.92		45.58		63.43		50.1			<.001	
	Beijing					106.20		58.92		73.68		119.28		173.59		45.1					
	Tianjin					50.27		26.08		33.13		59.15		82.68		49.8					
	Hebei					27.23		12.67		17.23		33.90		44.83		56.3					
	Shanxi					27.42		13.06		18.21		32.62		45.56		54.2					
	Inner Mongolia					24.75		12.84		17.64		28.39		40.02		47.5					
**Northeast China**							30.23		15.95		19.98		34.36		50.84		49.5			<.001	
		Heilongjiang				24.69		13.57		16.15		28.27		40.98		47.3					
		Jilin				29.14		14.79		19.86		33.87		48.32		50.5					
		Liaoning				35.70		18.75		23.38		39.92		60.90		50.2					
**East China**							47.44		25.78		32.63		53.15		77.62		46.1			<.001	
		Shanghai				103.32		56.09		70.14		116.35		170.43		46.8					
		Jiangsu				56.48		30.07		39.37		63.74		92.43		47.0					
		Zhejiang				67.79		38.87		48.80		73.54		108.15		41.6					
		Anhui				27.69		14.55		18.84		30.66		46.22		48.5					
		Jiangxi				26.02		14.03		17.79		29.22		42.80		46.8					
		Shandong				35.80		18.08		23.59		42.03		59.24		51.3					
		Fujian				51.42		30.76		34.98		55.58		83.63		41.4					
**Central China**							29.71		14.97		20.37		33.5		49.77		50.7			<.001	
				Henan			27.07		13.23		17.87		31.55		45.40		53.2				
				Hubei			35.28		19.34		24.28		39.18		58.17		46.0				
				Hunan			28.60		13.63		20.49		31.35		48.64		52.8				
**South China**							50.85		28.45		34.04		56.75		82.97		45.1			<.001	
	Guangdong					60.10		34.28		39.62		66.73		98.07		44.4					
	Guangxi					31.81		16.97		22.75		35.49		51.55		45.9					
	Hainan					39.37		19.04		26.34		47.60		63.75		52.5					
**Southwest China**							35.25		17.23		23.45		39.84		60.05		53.3			<.001	
			Chongqing				44.75		22.05		29.40		49.10		77.67		53.6				
			Sichuan				39.32		19.04		25.18		44.69		68.01		55.2				
			Guizhou				25.04		11.66		17.25		28.77		42.17		54.8				
			Yunnan				30.35		15.51		21.51		34.57		49.46		48.5				
			Tibet				26.51		12.07		20.14		28.36		44.44		53.0				
**Northwest China**							24.91		13.15		17.9		28.51		39.71		46.0			<.001	
					Shaanxi		32.25		15.98		21.83		37.84		52.99		51.4				
					Ningxia		24.94		12.40		17.75		29.57		39.59		49.1				
					Qinghai		20.97		9.76		16.19		25.00		32.62		50.0				
					Xinjiang		20.13		13.42		16.35		20.19		30.11		30.2				
					Gansu		19.56		9.75		14.02		23.22		31.09		49.0				

^a^HIVSTKPP: HIV self-testing kit–purchasing population.

^b^The average annual percentage change from January 1, 2016, to December 31, 2019, was calculated by joinpoint regression model.

**The top 10 cities with the highest average annual HIV self-testing kit–purchasing population (HIVSTKPP) rates.**

**Top 10 cities with the highest average HIVSTKPP rates (per 100,000 population)**

- Shenzhen in Guangdong province: 107.92
- Hangzhou in Zhejiang province: 97.17
- Zhuhai in Guangdong province: 93.98
- Nanjing in Jiangsu province: 86.75
- Guangzhou in Guangdong province: 85.74
- Beijing: 83.86
- Shanghai: 80.88
- Chengdu in Sichuan province: 80.62
- Xiamen in Fujian province: 80.49
- Wuhan in Hubei province: 79.37

**The top 10 cities with the largest sizes of HIV self-testing kit–purchasing population (HIVSTKPP).**

**Top 10 cities with the largest sizes of HIVSTKPP**

- Shanghai: 0.10 million
- Beijing: 0.09 million
- Shenzhen in Guangdong province: 0.07 million
- Chengdu in Sichuan province: 0.07 million
- Guangzhou in Guangdong province: 0.07 million
- Chongqing: 0.06 million
- Hangzhou in Zhejiang province: 0.05 million
- Wuhan in Hubei province: 0.05 million
- Suzhou in Jiangsu province: 0.04 million
- Nanjing in Jiangsu province: 0.04 million

### Spatial Autocorrelation Patterns

The global Moran I showed significant positive spatial clustering of average annual HIVSTKPP rates at the city and provincial-controlled–county levels during the entire study period (Moran I=0.252; *P*<.001; Table S1 in Multimedia Appendix 2). The global Moran I decreased from 0.317 in 2016 to 0.234 in 2019. Significant local indicators of spatial association were mainly detected in high-high and low-low clusters (Table 4).

**Figure 14:**
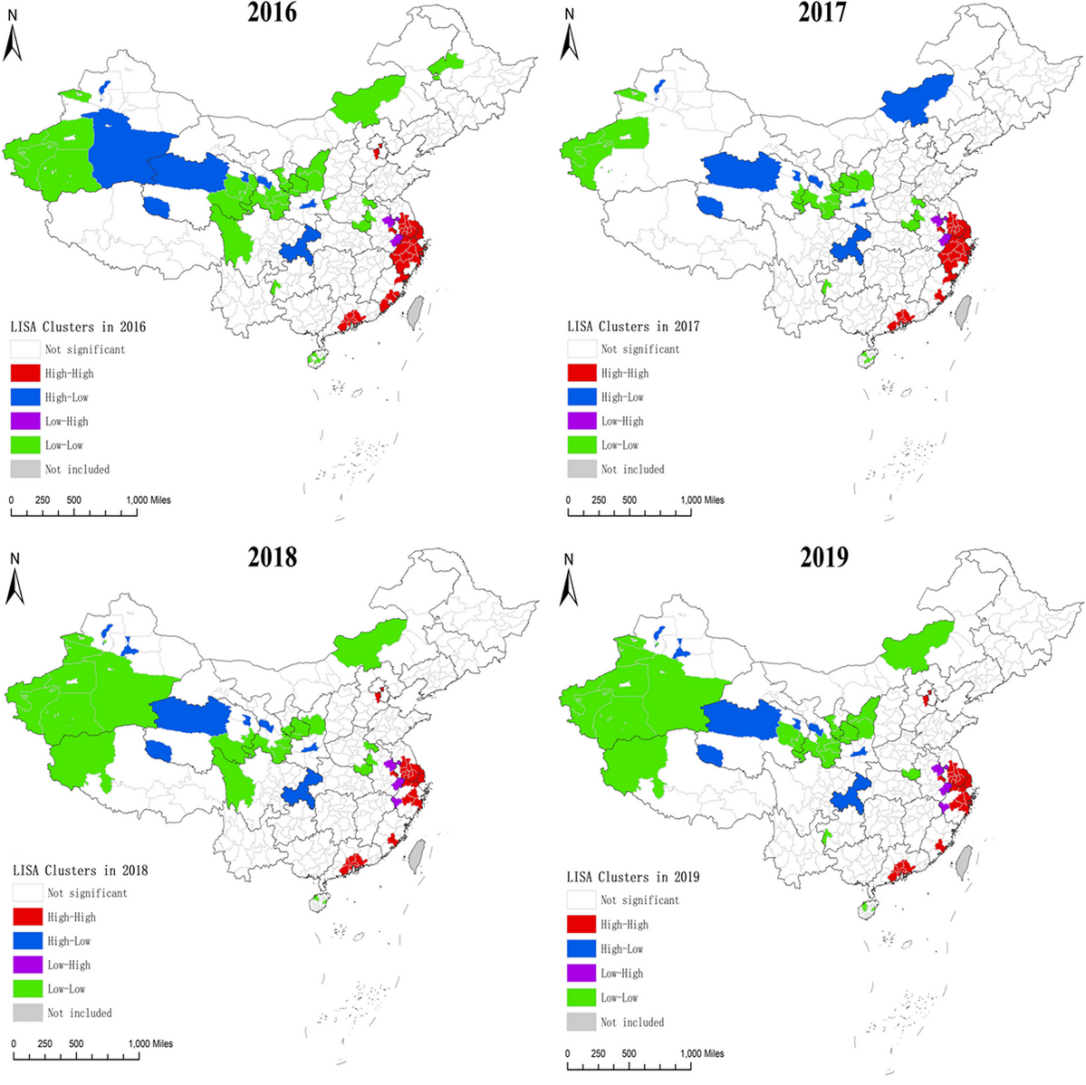
Geographical distribution of local clusters of the population-level HIV self-testing kit–purchasing population rate per 100,000 population from January 1, 2016, to December 31, 2019, at city and provincial-controlled–county levels in mainland China by spatial autocorrelation analysis. LISA: local indicators of spatial association.

Between January 1, 2016, and December 31, 2019, of the 366 cities and provincial-controlled counties, 26 (7.1%) and 27 (7.4%) were identified as high-high and low-low clusters, respectively (Table 4). The average annual HIVSTKPP rate in high-high clusters (79.63 per 100,000) was 5.29-fold higher than that in low-low clusters (15.06 per 100,000). The high-high clusters, where the HIVSTKPP accounted for 26.16% (570,261/2,180,284) of all anonymous persons, were mainly located in East and South China, including Zhejiang, Guangdong, and Jiangsu provinces (Figure 12B and Table S2 in Multimedia Appendix 2). From January 1, 2016, to December 31, 2019, the local indicators of spatial association clusters remained relatively stable (Figure 14). The number of cities in high-high clusters decreased from 31 in 2016 to 24 in 2019, and the proportion of HIVSTKPP (high-high clusters vs all anonymous persons) decreased from 28.16% (80,297/285,107) in 2016 to 23.89% (216,848/907,539) in 2019, whereas the HIVSTKPP rates significantly increased from 41.61 per 100,000 in 2016 to 124.93 per 100,000 in 2019 (AAPC=45.6%; *P*<.001; Table 4).

**Table 4:** Descriptive statistics of 4 types of spatial clusters as defined by local indicators of spatial association analysis between January 1, 2016, and December 31, 2019, in mainland China.

Period and patterns		Rate^a^	HIV self-testing kit–purchasing population, n (%)	Cities (N=366), n (%)
**January 1, 2016, to December 31, 2019 (N=2,180,284)**				
	High-high	79.63	570,261 (26.16)	26 (7.10)
	High-low	52.01	100,477 (4.61)	7 (1.91)
	Low-high	22.53	6062 (0.28)	2 (0.55)
	Low-low	15.06	34,220 (1.57)	27 (7.38)
**2016 (N=285,107)**				
	High-high	41.61	80,297 (28.16)	31 (8.47)
	High-low	26.34	12,377 (4.34)	7 (1.91)
	Low-high	13.00	864 (0.30)	2 (0.55)
	Low-low	7.65	5522 (1.94)	34 (9.29)
**2017 (N=369,140)**				
	High-high	54.38	95,153 (25.78)	27 (7.38)
	High-low	34.94	16,744 (4.54)	7 (1.91)
	Low-high	15.80	1057 (0.29)	2 (0.55)
	Low-low	10.80	5396 (1.46)	22 (6.01)
**2018 (N=618,498)**				
	High-high	86.16	140,666 (22.74)	23 (6.28)
	High-low	60.35	30,046 (4.86)	7 (1.91)
	Low-high	26.54	2381 (0.38)	3 (0.82)
	Low-low	16.91	9840 (1.59)	27 (7.38)
**2019 (N=907,539)**				
	High-high	124.93	216,848 (23.89)	24 (6.56)
	High-low	89.98	45,060 (4.97)	7 (1.91)
	Low-high	38.68	3491 (0.38)	3 (0.82)
	Low-low	22.16	11,617 (1.28)	29 (7.92)

^a^The rate of HIV self-testing kit purchasing per 100,000 population was calculated by dividing the number of purchasers by the total population in an area.

### Spatiotemporal Clusters

Clusters with higher demand for HIVST kits available on the web were detected in 75 cities and provincial-controlled counties located in East China, South China, and Central China between January 1, 2018, and December 31, 2019, including 15 (20%) in Guangdong province, 11 (15%) in Zhejiang province, 11 (15%) in Jiangxi province, 10 (13%) in Anhui province, 9 (12%) in Fujian province, 7 (10%) in Jiangsu province, 6 (8%) in Hunan province, 5 (7%) in Hubei province, and 1 (1%) in Shanghai. Clusters with lower demand for HIVST kits available on the web were found in 181 cities and provincial-controlled counties between January 1, 2016, and December 31, 2017 (Figure 12C and Table 5). High-demand and low-demand clusters accounted for 42.2% (643,977/1,526,012) and 29.74% (194,571/654,240) of the total HIVSTKPP, respectively. The HIVSTKPP rate in high-demand clusters (165.79 per 100,000) was 5.03-fold higher than in low-demand clusters (32.94 per 100,000). Compared with neighboring cities and provincial-controlled counties, those identified in high-demand clusters had 2.55 times more HIVSTKPP (relative risk=2.55; *P*<.001).

**Table 5:** General description of the high-risk and low-risk clusters identified by temporal-spatial clustering analysis between January 1, 2016, and December 31, 2019, in mainland China.

Cluster type	Time interval	Cluster center (latitude, longitude)	Radius (km)	Cities (N=366), n (%)	Provinces included (number of cities)	Purchasers (N=2,180,284), n (%)	Rate^a^	Relative risk	Log likelihood ratio	*P* value
High-risk cluster	January 1, 2018, to December 31, 2019	26.049704 N, 119.180295 E	731.09	75 (20.49)	Guangdong (15), Zhejiang (11), Jiangxi (11), Anhui (10), Fujian (9), Jiangsu (7), Hunan (6), Hubei (5), and Shanghai	643,977 (42.20)	165.79	2.55	171422.12	<.001
Low-risk cluster	January 1, 2016, to December 31, 2017	36.712360 N, 94.531065 E	1931.67	181 (49.45)	Xinjiang (24), Sichuan (21), Henan (18), Yunnan (16), Gansu (14), Hubei (12), Shanxi (11), Shaanxi (10), Guizhou (9), Qinghai (8), Hunan (7), Inner Mongolia (7), Tibet (7), Hebei (6), Ningxia (5), Guangxi (3), Shandong (2), and Chongqing	194,571 (29.74)	32.94	0.36	119468.41	<.001

^a^The rate of HIV self-testing kit purchasing per 100,000 population was calculated by dividing the number of purchasers by the total population in an area.

### Spatiotemporal Patterns

**Figure 15:**
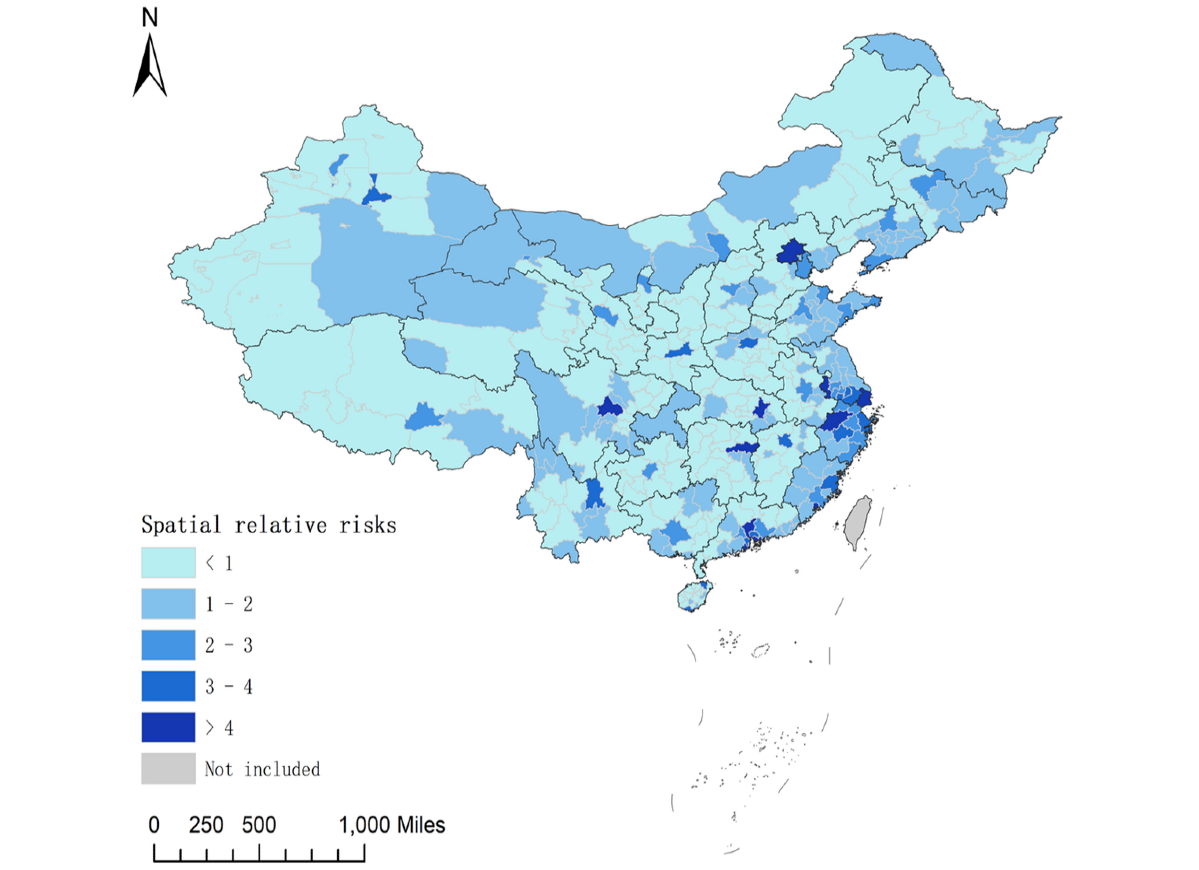
Geographical distribution of relative risk of the spatial effect at city and provincial-controlled–county levels from January 1, 2016, to December 31, 2019, in mainland China through the best-fitting Bayesian spatiotemporal model.

We found that the spatiotemporal model with type IV interaction had the lowest deviance information criterion valve. Therefore, this was the best-fit model and was applied in our analysis (Table S3 in Multimedia Appendix 2). During the study period, the highest spatial relative risk was noted in Shenzhen city of Guangdong province, Hangzhou city of Zhejiang province, and Zhuhai city of Guangdong province (Figure 15). Precisely 37.2% (136/366) and 48.1% (176/366) of the cities and provincial-controlled counties were classified as hot spots (higher demand for HIVST kits) and cold spots (lower demand for HIVST kits), respectively (Figure 12D and Table S4 in Multimedia Appendix 2).

**Figure 16:**
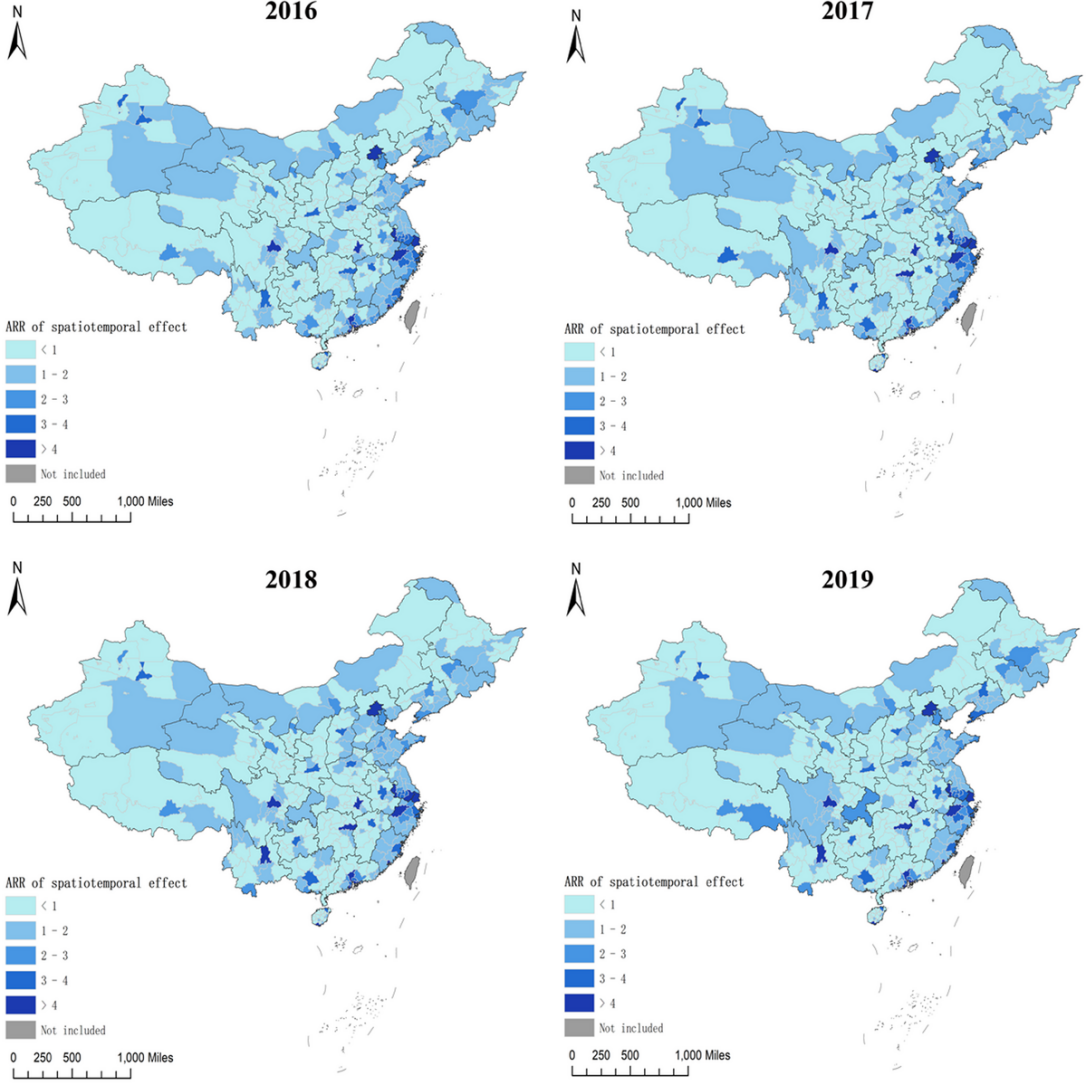
Geographical distribution of relative risk of the spatiotemporal interaction effect at city and provincial-controlled–county levels from January 1, 2016, to December 31, 2019, in mainland China through the best-fitting Bayesian spatiotemporal model. ARR: adjusted relative risk.

Spatial variations in adjusted relative risk (ARR; relative demand for HIVST kits) of spatiotemporal interaction effect from January 1, 2016, to December 31, 2019, are presented in Figure 16. The ARRs in each region between January 1, 2016, and December 31, 2019, varied slightly, and the higher ARR (higher demand for HIVST kits) continued to be applicable in municipalities, provincial capitals, and large cities.

### Correlates of HIVSTKPP

**Figure 17:**
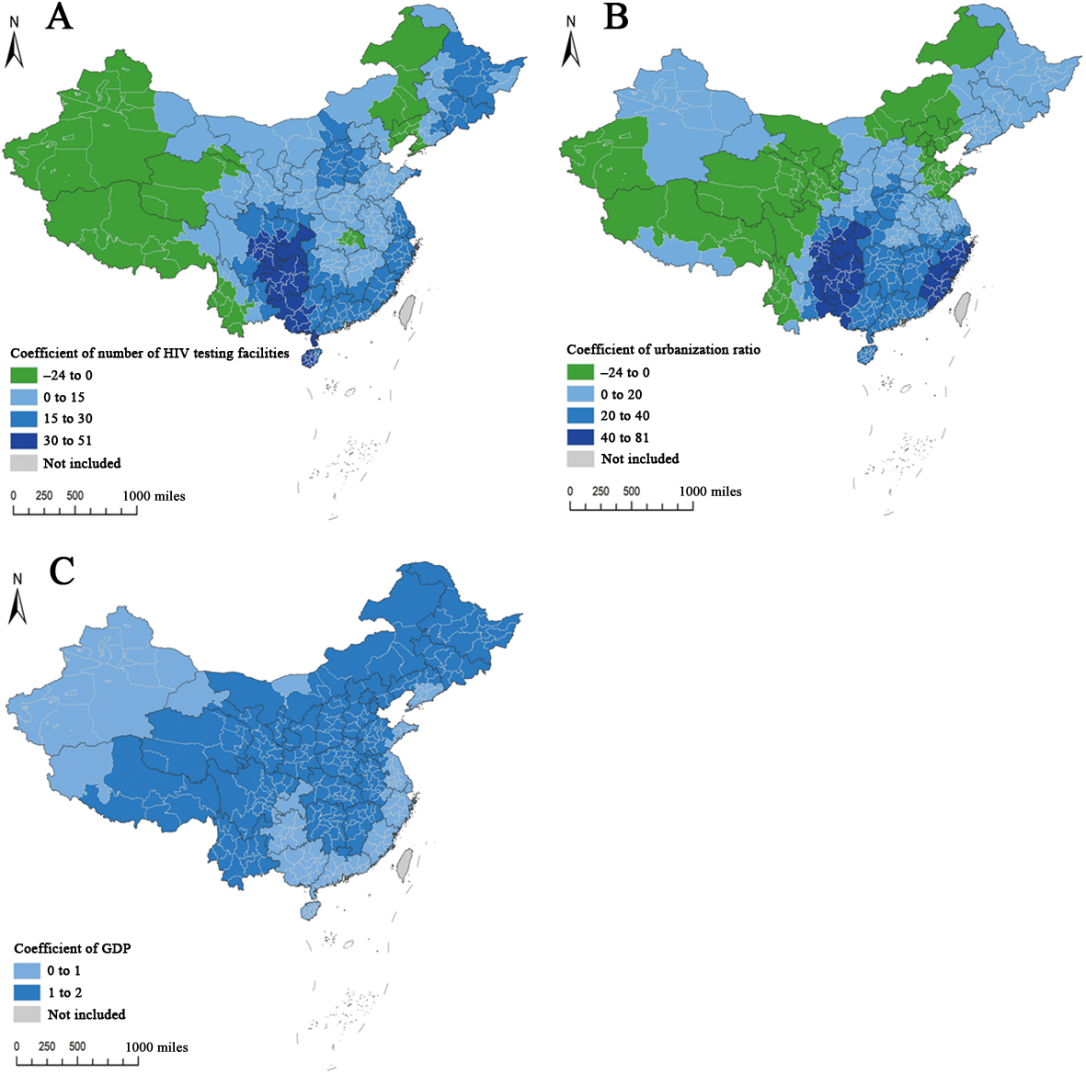
Geographical distribution of coefficients of the (A) number of HIV testing facilities, (B) urbanization ratio, and (C) gross domestic product (GDP) in the association with the number of the HIV self-testing kit–purchasing population at city and provincial-controlled–county levels in mainland China in 2019 through a geographically weighted regression model.

The SEM was found to be the most appropriate model for interpreting associations between city and provincial-controlled county–based factors and the HIVSTKPP (data not shown). The SEM results showed that the number of HIV testing facilities, urbanization ratio, and GDP per capita were positively associated with HIVSTKPP for the whole country, with the regression coefficients being 14.106, 11.236, and 1.057, respectively (all *P* values met the threshold for statistical significance; Table S5 in Multimedia Appendix 2). Notably, the geographically weighted regression model revealed a strong spatial heterogeneity in relationships between the HIVSTKPP and number of HIV testing facilities as well as urbanization ratio (Figure 17 and Table S6 in Multimedia Appendix 2). The number of HIV testing facilities and urbanization ratio were found to have a positive correlation with the HIVSTKPP in most cities (306/366, 83.6%, and 289/366, 79%, respectively). Cities with large positive correlations with the number of HIV testing facilities were concentrated in Sichuan and Guizhou provinces. In terms of urbanization ratio, cities with greater positive correlations were also predominantly in Sichuan and Guizhou provinces.

## Discussion

### Principal Findings

In this retrospective analysis of data involving 2.18 million anonymous persons in China who ordered 4.51 million HIVST kits on the web between January 1, 2016, and December 31, 2019, we identified key regions in which there was a larger demand for HIVST kits. Between January 1, 2016, and December 31, 2019, the size of the HIVSTKPP as well as HIVSTKPP rates increased across regions. High HIVSTKPP rates and hot spots identified by Bayesian spatiotemporal analysis were mainly located in large cities, provincial capitals, and municipalities. Spatial autocorrelation analysis identified high-high clusters and space-time cluster analysis identified higher-demand clusters that were predominantly in cities along China’s southeast coast. The number of HIV testing facilities, urbanization ratio, and GDP per capita were correlated with larger HIVSTKPP for the whole country.

### The Interpretation of the Temporal Distribution of the HIVSTKPP and Its Implication for HIV Prevention

We observed a steady increase in HIVST kit purchases over the 4-year study period. The growth in the size of the HIVSTKPP accelerated after 2017. This growth may have been prompted by the 13th Five-Year Plan for HIV Prevention and Control, which directed the CCDC and nongovernmental organizations to focus on promoting HIVST at the population level, including organizing a series of promotional activities for the *Nationwide HIV Testing and Consultation Month* program between November 20 and December 20 in both 2018 and 2019 to raise public awareness of HIV testing and promote active testing [40,41]. Annual increases in the HIVSTKPP could also be explained by the fact that people are increasingly relying on web-based purchasing for their shopping generally, and HIVST kits are no different than other products. However, the numbers of HIV tests and of newly diagnosed people living with HIV in China did not show a similar rate of growth over the same period. The total number of HIV tests conducted in China was 169 million, 200.72 million, and 240.87 million in 2016, 2017, and 2018, respectively, which corresponded to annual growth rates of 18.77% in 2017 and 20% in 2018 [5]. The total number of newly diagnosed people living with HIV in China was 124,555, 134,512, and 148,589 in 2016, 2017, and 2018 respectively, which corresponded to annual growth rates of 7.99% in 2017 and 10.46% in 2018 [5]. Both were dwarfed by the increases in the HIVSTKPP in our study, with 0.64 million, 0.83 million, and 1.20 million in 2016, 2017 and 2018, respectively, which corresponded to annual growth rates of 29.80% in 2017 and 44.51% in 2018. The ranking of the size of the HIVSTKPP among 31 provinces was also different from those of HIV tests and newly diagnosed people living with HIV [5]. Although our study is not able to tease out whether the observed widespread and rapidly growing uptake of web-based purchase of HIVST kits is purely coming from a diffusion process of innovation or compounded with a worsening underlying epidemic, these phenomena confirm the potential existence of a previously unmet need in HIV prevention and control and highlight the urgent need for a novel strategy that links the HIVSTKPP to facility-based HIV testing and care.

The maximum monthly number of web-based purchases of HIVST kits occurs in December, which might indicate the effectiveness of public education campaigns that promote HIV testing on World AIDS Day (December 1). However, efforts to contain the epidemic should be in place throughout the year. Besides World AIDS Day, prevention campaigns may take place on a more frequent and regular basis.

### The Interpretation of the Spatial Distribution of the HIVSTKPP and Its Implication for HIV Prevention

High HIVSTKPP rates and hot spots were identified in large cities, provincial capitals, and municipalities. The spatiotemporal distribution of the HIVSTKPP at local levels in our study provides policy makers with rare and valuable information about the number of people who are potentially in need of testing and treatment services. This may guide public health authorities to allocate resources, develop interventions, and deliver services.

Recent studies showed that HIV epidemic clusters among MSM spread from a few large cities in eastern China to most of the municipalities and provincial capitals countrywide between 2006 and 2015 [13]. HIV epidemic clusters among young people aged 15 to 24 years spread from southwestern China to central and northeastern China between 2005 and 2012 [14]. However, in our study, spatial clusters of the HIVSTKPP were mainly found in southeastern coastal cities between 2016 and 2019. Some studies showed that the majority of the HIVSTKPP reported that they had engaged in high-risk sexual behaviors in the last 6 months [12,42]. Thus, the web-based HIVST kit–purchasing behavior could largely indicate that recent high-risk sexual behavior may have been a factor in HIV acquisition, and the high-demand spatiotemporal cluster of the HIVSTKPP identified in our study could serve as an early warning sign for new HIV epidemics. Potential gaps in web-based HIVST kit–purchasing behavior and an HIV epidemic might exist and should be further identified by connecting and comparing our findings with these patterns of HIV epidemics in the same population and study period for a timely adjustment of the HIV prevention and control strategy.

### The Interpretation of the Associations Between HIVST Kit Purchasing and Macroscopic Factors and Its Implication for HIV Prevention

Our SEM identified positive correlations between a region’s number of HIV testing facilities, urbanization ratio, and GDP per capita and the HIVSTKPP. The number of HIV testing facilities was positively correlated with the size of the HIVSTKPP in 306 cities and provincial-controlled counties. Regions with larger numbers of HIV testing facilities may have higher levels of relevant health education, thus further promoting HIV testing. In addition, the urbanization ratio was positively associated with the size of the HIVSTKPP in 289 cities and provincial-controlled counties. The positive effect could be associated with the assumption that the increase in the proportion of the urban population may promote social networking and sexually risky behaviors (eg, multiple sexual partners, unprotected sex behaviors, and commercial sexual behaviors) [28], thus leading to an increase in the demand for HIVST kits. GDP per capita did not have an important role to play with regard to the size of the HIVSTKPP, and its effect varies slightly across regions. Thus, the spatial patterns of the HIVSTKPP could be largely explained by the spatial variation in the number of HIV testing facilities and urbanization ratio.

Our study shows more HIVST kit purchases in areas with greater access to HIV testing centers and more urbanization. For areas currently lacking adequate access to HIV testing, especially areas bearing heavy HIV burdens (eg, rural provinces such as Tibet, Sichuan, Guizhou, and Guangxi) [1,5,43], our study also found that there were few web-based HIVST kit purchases. Thus, public education campaigns that promote web-based HIVST kit purchases at the population level are urgently required to expand HIV testing as well as HIV prevention and control programs in these areas.

### Comparison With Prior Work

To the best of our knowledge, this study is the first to evaluate the spatiotemporal patterns of the HIVSTKPP. Monitoring HIVST kit purchasing patterns could be a new tool for public health policy makers, researchers, and program implementers to reallocate resources, promote HIVST uptake, and optimize the HIV care continuum.

### Limitations

Our study includes several limitations. First, although sales records from a leading e-commerce platform in China were included in our study, we did not include records from all e-commerce platforms, HIV clinics, hospitals, offline pharmacies, and CCDC offices. The sample in our study does not represent the entire HIVSTKPP in China, and HIVSTKPP rates are underestimated in our analysis. Our findings should be interpreted with caution, and further research that involves collecting data from all potential sources of HIVST kits is needed. Second, we could not quantitatively assess associations between the HIVSTKPP and HIV epidemiological data to identify potential gaps in current HIV-monitoring practices in China. In addition, we could not compare our findings with national CCDC data about new HIV diagnoses that were first screened using self-testing because these data were not publicly available, and we have no access to these data. Third, we could not evaluate the spatial patterns of the HIVSTKPP in finer geographic units (eg, street level). Finally, many sex workers, including male sex workers, could have purchased HIVST kits on the web in bulk at the end of the day. However, our study did not include the records of bulk purchases for analysis.

### Conclusions

Our study provides an understanding of the spatiotemporal patterns of the HIVSTKPP in mainland China, which can inform the development of national and local HIVST guidelines in allocating resources and promoting HIV testing. The development of contextualized prevention and intervention strategies tailored to the HIVSTKPP in key regions identified from our study is urgently required for HIV prevention and control. Further research combining the spatiotemporal patterns of HIVST with HIV epidemiological data is needed to identify potential gaps in current HIV-monitoring practices and develop comprehensive HIV control strategies.

## Appendix

Multimedia Appendix 1 Details of the statistical analysis.

Multimedia Appendix 2 Detailed results (Tables S1-S6) of spatiotemporal analysis of the HIV self-testing kit–purchasing population (HIVSTKPP) from January 1, 2016, to December 31, 2019 in mainland China.

## Abbreviations

AAPC: average annual percentage change
ARR: adjusted relative risk
CCDC: Chinese Center for Disease Control and Prevention
GDP: gross domestic product
HIVST: HIV self-testing
HIVSTKPP: HIV self-testing kit–purchasing population
MSM: men who have sex with men
SEM: spatial error model
