# Supplementary material for: Spatiotemporal Distribution of HIV Self-testing Kits Purchased on the Web and Implications for HIV Prevention in China: Population-Based Study
Source: JMIR Public Health Surveill. 2022 Oct 4;8(10):e35272. doi: 10.2196/35272 (PMC9579936; doi:10.2196/35272)
Supplement: Multimedia Appendix 1 [file publichealth_v8i10e35272_app1.docx]

Appendix 1

Statistical Analysis

Temporal Trend Analysis

We used a seasonal-trend decomposition procedure based on locally weighted regression (STL) to describe temporal trends in the monthly number of HIVST kit purchasers. Each time series was broken into overall trends, seasonal trends, and remainder components. The secular trends and seasonal patterns were depicted by the plot of decomposition, monthly and, seasonal distribution.

Average annual percent change (AAPC) were calculated using a Joinpoint regression model with natural log-transformed rates to assess changes in HIVSTKPP rates from 2016 to 2019 [1]. Trends were regarded as increasing or decreasing if the AAPC was statistically significantly different from zero ($P$-value < 0.05). Otherwise trends were described as stable [1]. HIVSTKPP rates were presented on a semi-log scale to contrast temporal trends and magnitude across regions [2]. We also drew the average annual HIVSTKPP rate during 2016-2019 versus AAPC on an arithmetic scale.

Spatial Autocorrelation Analysis

The spatial dependency and heterogeneity for annual HIVSTKPP rates at city/provincial-controlled county level was explored by spatial autocorrelation analysis. Global spatial autocorrelation was used to evaluate the overall spatial distribution patterns (cluster/disperse/random) throughout the country [3], and local spatial autocorrelation was used to identify specific local clusters [4]. Global Moran's I and local Moran's I were calculated for global and local spatial autocorrelation, respectively [5].

The value of global Moran's I ranged from -1 to +1, with positive values indicating spatial clustering of similar HIVSTKPP rates and negative values indicating spatial clustering of dissimilar HIVSTKPP rates. A Moran's I ≥ 0.2 was considered highly clustered [6]. Global Moran's I values were examined by Z scores, with Z > 1.96 or Z < -1.96 indicating statistically significant autocorrelation.

By calculating local Moran's I values comparing the HIVSTKPP rate in a given city to average rates in neighboring cities, four types of local spatial clusters were identified: high-high, low-low, high-low, and low-high [7]. High-high and low-low clusters indicate a city and its surrounding region jointly had a higher or lower HIVSTKPP rate than average, respectively. A high-low cluster represents a city with an above average HIVSTKPP rate surrounded by cities with below average rates, while a low-high cluster represents a city with a below average HIVSTKPP rate surrounded by cities with above average rates. The significance of clusters was examined by Z-test, with $P$-value < 0.05 indicating statistically significant local spatial autocorrelation. For both global and local Moran's I, Monte Carlo randomization (999 permutations) was applied to evaluate the test statistic. All spatial analyses in this study were conducted by first-order queen contiguity-based spatial weights.

Temporal-Spatial Clustering Analysis

Retrospective space-time scan analyses based on discrete Poisson distribution was performed to identify high-risk (higher demands) and low-risk (lower demands) clusters of HIVSTKPP at city/provincial-controlled county level over space and time simultaneously [8, 9]. This method was defined by a cylindrical scanning window, where the circular base denoted the area of the scanned space and the height represented the scanning time period [10]. The window was moved both in space and time to scan each time interval for each geographical region. The analysis was conducted using a one-year time interval, a maximum spatial cluster size of 50% of the at-risk population, and a maximum temporal cluster size of 50% of the study period [10]. The relative risk (RR) was obtained by the ratio of the observed number of HIVSTKPP to the expected number of HIVSTKPP inside and outside the window. Log likelihood ratios (LLR) were calculated by a likelihood function [10] to compare risk outside and inside the window. Statistical significance of the spatiotemporal clusters was evaluated using Monte Carlo simulations with 999 replications, with P-value < 0.05 set as threshold for statistical significance.

Bayesian Spatiotemporal Model

A Bayesian spatiotemporal model was used to calculate spatial RR. The HIVSTKPP was assumed to follow a Poisson distribution. Then, six models based on log-linear regression functions were constructed as follows:

Model 1: The Besag–York–Molliè (BYM) model [11] was extended to include a linear term for a parametric trend for the temporal component [12]. The form of the first model was specified as follows:

$$y_{ij}\sim Poisson(\lambda_{ij})$$

$$E\left( y_{ij} \right)=\lambda_{ij}=e_{ij}\times\theta_{ij}$$

$$\log\left( \theta_{ij} \right)=b_{0}+u_{i}+\nu_{i}+(\alpha+\beta_{i})\times j$$

Model 2: The BYM model can also be extended to include a linear term for a dynamic nonparametric spatiotemporal time trend [13]. The form of the second model was as follows:

$$\log\left( \theta_{ij} \right)=b_{0}+u_{i}+\nu_{i}+\gamma_{j}+\phi_{j}$$

Model 3: The above model can be expanded to allow for an interaction between space and time [13]. The form of the third model with type I interaction was as follows:

$$\log\left( \theta_{ij} \right)=b_{0}+u_{i}+\nu_{i}+\gamma_{j}+\phi_{j}+\delta_{ij1}$$

Model 4: The form of the fourth model with type II interaction [13] was as follows:

$$\log\left( \theta_{ij} \right)=b_{0}+u_{i}+\nu_{i}+\gamma_{j}+\phi_{j}+\delta_{ij2}$$

Model 5: The form of the fifth model with type III interaction [13] was as follows:

$$\log\left( \theta_{ij} \right)=b_{0}+u_{i}+\nu_{i}+\gamma_{j}+\phi_{j}+\delta_{ij3}$$

Model 6: The form of the sixth model with type IV interaction [13] was as follows:

$$\log\left( \theta_{ij} \right)=b_{0}+u_{i}+\nu_{i}+\gamma_{j}+\phi_{j}+\delta_{ij4}$$

Among above models, $i$ ($i$ = 1, 2,…, 366) is an index for the spatial units (city and provincial-controlled county), and $j$ ($j$ = 1, 2, 3, 4) is an index for the time period (year); $y_{ij}$ is the observed HIVSTKPP in region $i$ during year $j$; $y_{ij}$ was assumed to follow a Poisson distribution with a mean $\lambda_{ij}=e_{ij}\times\theta_{ij}$; $e_{ij}$ is the expected HIVSTKPP in region $i$ during year $j$, which was calculated as the product of overall rate of the whole country and the population for each region during the study period; $\theta_{ij}$ is the spatiotemporal-specific RR, corresponding to the ratio of observed HIVSTKPP to expected HIVSTKPP of each region in each year; $b_{0}$ is the intercept, quantifying the average incidence RR in the whole country; $u_{i}$ and $\nu_{i}$ are the spatially structured and unstructured random effect, denoting that region $i$ has a similar pattern and an independent pattern of incidence with the adjacent regions, respectively; $\alpha$ is the main linear trend, indicating the global time effect; $\beta_{i}$ is a differential trend, identifying the interaction between time and space and illustrating the difference between the global trend and region-specific trends; $\gamma_{j}$ and $\phi_{j}$ are temporally structured and unstructured random effects, respectively; $\delta_{ij}$ is the interaction of the spatiotemporal effect, explaining differences in the temporal trend of HIVSTKPP rates from 2016 to 2019 for different cities, which includes type I interaction $\delta_{ij1}$ of the two unstructured effects, type II interaction $\delta_{ij2}$ of the structured temporal effect and unstructured spatially effect, type III interaction $\delta_{ij3}$ of the unstructured temporal effect and structured spatially effect, and type IV interaction $\delta_{ij4}$ of the two structured effects.

The default minimally informative priors were specified [14]. The spatial structured eﬀect was modeled using an intrinsic conditional autoregressive structure, while the temporally structured eﬀect was modeled dynamically through a time neighboring structure. The unstructured spatial and temporal eﬀects were both specified by Gaussian models with a mean of zero. The Gamma (1, 0.0005) was chosen as the prior for the precision of the above Gaussian random eﬀects. All Bayesian spatiotemporal models were fitted using the integrated nested Laplace approximation (INLA) method [15]. The model with the smallest deviance information criterion (DIC) was determined to have achieved the best balance between model complexity and fit [16], and was used in our study.

The region-specific spatial relative demands of HIVSTKPP compared to the whole country were calculated by performing an exponential transformation to the components of $u_{i}$ and $\nu_{i}$. To examine temporal changes of demands for HIVST in the geographical profile, the adjusted relative risk (ARR) proposed by Knorr-Held (2000) was measured by the exponentiation of the term $u_{i}$, $\nu_{i}$ and $\delta_{ij}$, namely$\mathrm{ARR}=exp(u_{i}+\nu_{i}+\delta_{ij})$ [13, 17, 18].

A city was determined to be a hot/cold spot for HIVST if it has a persistently higher/lower risk than overall level of the whole country. All cities were classified by the following criteria [19]. A city was identified as a hotspot if the posterior probability $p \left( \exp(u_{i}+\nu_{i} \right)>1 | data)$ was > 0.8. A city was regarded as a cold spot if the posterior probability $p \left( \exp(u_{i}+\nu_{i} \right)>1 | data)$ was < 0.2.

Spatial Regression Analysis

Spatial lag models (SLM), spatial error models (SEM), and geographically weighted regression (GWR) models were performed to examine the globally and locally spatial correlation between HIVSTKPP and three macroscopic factors in 2019. These factors, which are available at city/provincial-controlled county level, included (1) number of HIV testing facilities, (2) urbanization ratio, and (3) GDP per capita (10,000 Yuan/population). We used an ordinary least squares (OLS) regression model to determine spatial dependence and then selected the best fitting model by comparing the goodness-of-fit of the SLM and SEM [7]. Coefficient values for each factor at different spatial sites were obtained by detecting the spatial variation in relationships between HIVSTKPP and each macroscopic factors and then calculating the local parameters using the GWR model [20].

Statistical software

Joinpoint regression models were performed by Joinpoint Regression Program (version 4.8.0.1; IMS, Inc., Calverton, MD, USA). Spatial autocorrelation analyses, OLS, SLM, and SEM models were performed by GeoDa software (version 1.6.7; The Center for Spatial Data Science in the University of Chicago, Chicago, IL, USA). Temporal-spatial clustering analyses were performed by SaTScan software (version 9.6; Martin Kulldorff together with information Management Services Inc., Boston, USA). Trend surface analyses, Bayesian spatiotemporal models, and GWR models were conducted in R software (version 3.6.0; R Core Team, Vienna, Austria). Figures and maps were plotted by SigmaPlot software (version 12.5; SY Software, San Jose, Calif) and R software (version 3.6.0; R Core Team, Vienna, Austria), respectively.

**References**

1. Kim HJ, Fay MP, Feuer EJ, Midthune DN. Permutation tests for joinpoint regression with applications to cancer rates. Statistics in medicine. 2000 Feb 15;19(3):335-51. PMID: 10649300.

2. Devesa SS, Donaldson J, Fears T. Graphical presentation of trends in rates. American journal of epidemiology. 1995 Feb 15;141(4):300-4. PMID: 7840107.

3. Moran PA. Notes on continuous stochastic phenomena. Biometrika. 1950 Jun;37(1-2):17-23. PMID: 15420245.

4. Anselin L. Local indicators of spatial association—LISA. *Geographical analysis*. 1995;27(2):93-115. doi: 10.1111/j.1538-4632.1995.tb00338.x.

5. Peng ZH, Cheng YJ, Reilly KH, Wang L, Qin QQ, Ding ZW, et al. Spatial distribution of HIV/AIDS in Yunnan province, People's Republic of China. Geospatial health. 2011 May;5(2):177-82. PMID: 21590667. doi: 10.4081/gh.2011.169.

6. Wilt GE, Adams EE, Thomas E, Ekperi L, LeBlanc TT, Dunn I, et al. A space time analysis evaluating the impact of hurricane sandy on HIV testing rates. International journal of disaster risk reduction. 2018 2018/06/01/;28:839-44. doi: <https://doi.org/10.1016/j.ijdrr.2018.04.003>.

7. Qin Q, Guo W, Tang W, Mahapatra T, Wang L, Zhang N, et al. Spatial analysis of the human immunodeficiency virus epidemic among men who have sex with men in China, 2006-2015. Clinical infectious diseases : an official publication of the Infectious Diseases Society of America. 2017 Apr 1;64(7):956-63. PMID: 28362948. doi: 10.1093/cid/cix031.

8. Kulldorff M, Nagarwalla N. Spatial disease clusters: detection and inference. Statistics in medicine. 1995 Apr 30;14(8):799-810. PMID: 7644860. doi: 10.1002/sim.4780140809.

9. Kulldorff M, Athas WF, Feurer EJ, et al. Evaluating cluster alarms: a space-time scan statistic and brain cancer in Los Alamos, New Mexico. American journal of public health. 1998 Sep;88(9):1377-80. PMID: 9736881. doi: 10.2105/ajph.88.9.1377.

10. Zhang X, Tang W, Li Y, Mahapatra T, Feng Y, Li M, et al. The HIV/AIDS epidemic among young people in China between 2005 and 2012: results of a spatial temporal analysis. HIV medicine. 2017 Mar;18(3):141-50. PMID: 27552922. doi: 10.1111/hiv.12408.

11. Besag J, York J, Mollié A. Bayesian image restoration, with two applications in spatial statistics. Annals of the Institute of Statistical Mathematics. 1991 1991/03/01;43(1):1-20. doi: 10.1007/BF00116466.

12. Bernardinelli L, Clayton D, Pascutto C, Montomoli C, Ghislandi M, Songini M. Bayesian analysis of space-time variation in disease risk. Statistics in medicine. 1995 Nov 15-30;14(21-22):2433-43. PMID: 8711279. doi: 10.1002/sim.4780142112.

13. Knorr-Held L. Bayesian modelling of inseparable space-time variation in disease risk. Statistics in medicine. 2000 Sep 15-30;19(17-18):2555-67. PMID: 10960871. doi: 10.1002/1097-0258(20000915/30)19:17/18<2555::aid-sim587>3.0.co;2-#.

14. Blangiardo M, Cameletti M, Baio G, Rue H. Spatial and spatio-temporal models with R-INLA. Spatial and spatio-temporal epidemiology. 2013 Dec;7:39-55. PMID: 24377114. doi: 10.1016/j.sste.2013.07.003.

15. Rue H, Martino S, Chopin N. Approximate Bayesian inference for latent Gaussian models by using integrated nested Laplace approximations. 2009;71(2):319-92. doi: 10.1111/j.1467-9868.2008.00700.x.

16. Spiegelhalter DJ, Best NG, Carlin BP, Van Der Linde A. Bayesian measures of model complexity and fit. 2002;64(4):583-639. doi: 10.1111/1467-9868.00353.

17. Papoila AL, Riebler A, Amaral-Turkman A, São-João R, Ribeiro C, Geraldes C, et al. Stomach cancer incidence in Southern Portugal 1998-2006: a spatio-temporal analysis. Biometrical journal Biometrische Zeitschrift. 2014 May;56(3):403-15. PMID: 24596314. doi: 10.1002/bimj.201200264.

18. Etxeberria J, Goicoa T, López-Abente G, et al. Spatial gender-age-period-cohort analysis of pancreatic cancer mortality in Spain (1990-2013). PloS one. 2017;12(2):e0169751. PMID: 28199327. doi: 10.1371/journal.pone.0169751.

19. Richardson S, Thomson A, Best N, et al. Interpreting posterior relative risk estimates in disease-mapping studies. Environmental health perspectives. 2004 Jun;112(9):1016-25. PMID: 15198922. doi: 10.1289/ehp.6740.

20. Wang Y, Zhao C, Liu Z, et al. Spatiotemporal analysis of AIDS incidence and its influencing factors on the Chinese mainland, 2005-2017. International journal of environmental research and public health. 2021 Jan 25;18(3). PMID: 33503938. doi: 10.3390/ijerph18031043.
