# Supplementary material for: Spatiotemporal Distribution of HIV Self-testing Kits Purchased on the Web and Implications for HIV Prevention in China: Population-Based Study
Source: JMIR Public Health Surveill. 2022 Oct 4;8(10):e35272. doi: 10.2196/35272 (PMC9579936; doi:10.2196/35272)
Supplement: Multimedia Appendix 2 [file publichealth_v8i10e35272_app2.docx]

| Table S1. Global Moran’s I statistics of HIV self-testing kit purchasing population rates per 100,000 population by spatial autocorrelation analysis during 2016-2019 in mainland China | | | | |
| --- | --- | --- | --- | --- |
| Year | Moran’s I | Z-score | P-value | Pattern |
| 2016-2019 | 0.2523 | 7.5167 | <0.001 | Cluster |
| 2016 | 0.3171 | 9.5115 | <0.001 | Cluster |
| 2017 | 0.2396 | 7.3079 | <0.001 | Cluster |
| 2018 | 0.2389 | 7.1213 | <0.001 | Cluster |
| 2019 | 0.2340 | 7.0602 | <0.001 | Cluster |

| Table S2. The detailed cities of High-High clusters as defined by a local indicators of spatial association analysis during 2016-2019 in mainland China | | | | | | | |
| --- | --- | --- | --- | --- | --- | --- | --- |
| Year | North China | Northeast China | East China | Central China | South China | Southwest China | Northwest China |
| 2016-2019 | Hebei Province: Langfang. | None | (1) Zhejiang Province: Hangzhou, Ningbo, Jinhua, Jiaxing, Zhoushan, Taizhou, Shaoxing, Huzhou, Quzhou;  (2) Shanghai;  (3) Jiangsu Province: Suzhou, Wuxi, Changzhou, Zhenjiang, Nantong, Taizhou;  (4) Fujian Province: Quanzhou;  (5) Anhui Province: Maanshan. | None | Guangdong Province: Shenzhen, Guangzhou, Dongguan, Zhongshan, Foshan, Huizhou, Jiangmen. | None | None |
|  |  |  |  |  |  |  |  |
| 2016 | Hebei Province: Langfang. | None | (1) Zhejiang Province: Hangzhou, Ningbo, Jinhua, Jiaxing, Zhoushan, Taizhou, Shaoxing, Huzhou, Lishui, Quzhou;  (2) Shanghai;  (3) Jiangsu Province: Suzhou, Wuxi, Changzhou, Zhenjiang, Yangzhou, Nantong, Taizhou;  (4) Fujian Province: Quanzhou, Putian, Ningde, Zhangzhou;  (5) Anhui Province: Maanshan. | None | Guangdong Province: Shenzhen, Guangzhou, Dongguan, Zhongshan, Foshan, Huizhou, Jiangmen. | None | None |
|  |  |  |  |  |  |  |  |
| 2017 | None | None | (1) Zhejiang Province: Hangzhou, Ningbo, Jinhua, Jiaxing, Zhoushan, Taizhou, Shaoxing, Huzhou, Lishui, Quzhou;  (2) Shanghai;  (3) Jiangsu Province: Suzhou, Wuxi, Changzhou, Zhenjiang, Yangzhou, Nantong, Taizhou;  (4) Fujian Province: Quanzhou, Ningde;  (5) Anhui Province: Maanshan. | None | Guangdong Province: Shenzhen, Guangzhou, Dongguan, Zhongshan, Huizhou, Jiangmen. | None | None |
|  |  |  |  |  |  |  |  |
| 2018 | Hebei Province: Langfang. | None | (1) Zhejiang Province: Jinhua, Jiaxing, Taizhou, Shaoxing, Huzhou;  (2) Shanghai;  (3) Jiangsu Province: Suzhou, Wuxi, Changzhou, Zhenjiang, Yangzhou, Nantong, Taizhou;  (4) Fujian Province: Quanzhou;  (5) Anhui Province: Maanshan. | None | Guangdong Province: Shenzhen, Guangzhou, Dongguan, Zhongshan, Foshan, Huizhou, Jiangmen. | None | None |
|  |  |  |  |  |  |  |  |
| 2019 | Hebei Province: Langfang. | None | (1) Zhejiang Province: Ningbo, Jinhua, Jiaxing, Shaoxing, Huzhou, Taizhou;  (2) Shanghai;  (3) Jiangsu Province: Suzhou, Wuxi, Changzhou, Zhenjiang, Yangzhou, Nantong, Taizhou;  (4) Fujian Province: Quanzhou;  (5) Anhui Province: Maanshan. | None | Guangdong Province: Shenzhen, Guangzhou, Dongguan, Zhongshan, Foshan, Huizhou, Jiangmen. | None | None |

| Table S3. Deviance information criterion (DIC) for the selection of Bayesian spatiotemporal models during 2016-2019 in mainland China | | |
| --- | --- | --- |
| Model | Component | DIC  for general population |
| 1 | $\log\left( \theta_{ij} \right)=b_{0}+u_{i}+\nu_{i}+(\alpha+\beta_{i})\times j$ | 16807.08 |
| 2 | $\log\left( \theta_{ij} \right)=b_{0}+u_{i}+\nu_{i}+\gamma_{j}+\phi_{j}$ | 20330.98 |
| 3 | $\log\left( \theta_{ij} \right)=b_{0}+u_{i}+\nu_{i}+\gamma_{j}+\phi_{j}+\delta_{ij1}$ | 14585.28 |
| 4 | $\log\left( \theta_{ij} \right)=b_{0}+u_{i}+\nu_{i}+\gamma_{j}+\phi_{j}+\delta_{ij2}$ | 14542.9 |
| 5 | $\log\left( \theta_{ij} \right)=b_{0}+u_{i}+\nu_{i}+\gamma_{j}+\phi_{j}+\delta_{ij3}$ | 14474.48 |
| 6 | $\log\left( \theta_{ij} \right)=b_{0}+u_{i}+\nu_{i}+\gamma_{j}+\phi_{j}+\delta_{ij4}$ | 14443.39 |

| Table S4. Hot spots and cold spots identified by the best-fitting Bayesian spatiotemporal model during 2016-2019 in mainland China | | | | |
| --- | --- | --- | --- | --- |
| Hot/Cold spot | Number of cities (%) | Provinces included  (Number of cities) | Number of purchasers (%) | Rate^#^ |
| Hot spots | 136  (37.16) | Anhui (9), Beijing, Fujian (8), Gansu (3), Guangdong (10), Guangxi (5), Guizhou (1), Hainan (2), Hebei (8), Henan (6), Heilongjiang (5), Hubei (3), Hunan (3), Jilin (3), Jiangsu (11), Jiangxi (5), Liaoning (10), Inner Mongolia (8), Ningxia (1), Qinghai (2), Shandong (13), Shanxi (4), Shaanxi (1), Shanghai, Sichuan (4), Tianjin, Tibet (1), Xinjiang (5), Yunnan (5), Zhejiang (11), Chongqing. | 1,658,362  (76.06) | 61.06 |
|  |  |  |  |  |
| Cold spots | 176  (48.09) | Anhui (9), Gansu (10), Guangdong (6), Guangxi (7), Guizhou (7), Hainan (13), Hebei (5), Henan (10), Heilongjiang (6), Hubei (10), Hunan (11), Jilin (3), Jiangxi (5), Liaoning (4), Inner Mongolia (5), Ningxia (4), Qinghai (6), Shandong (3), Shanxi (5), Shaanxi (8), Sichuan (9), Tibet (5), Xinjiang (18), Yunnan (7). | 362,422  (16.62) | 16.80 |
| ^#^The rate of HIV self-testing kit purchasing per 100,000 population was calculated by dividing the number of purchasers by the total population in an area. | | | | |

| Table S5. Result of spatial error model among HIV self-testing kit purchasing population in 2019 in mainland China | | | |
| --- | --- | --- | --- |
| Variable | Coefficient | $z$ Value | $P$ |
| $\lambda$^a^ | 0.419 | 6.550 | <0.001 |
| Number of HIV testing facilities | 14.106 | 4.540 | <0.001 |
| Urbanization ratio | 11.236 | 2.136 | 0.0327 |
| GDP | 1.057 | 50.484 | <0.001 |
| $R^{2}$ | 0.946 |  |  |
| AIC | 6204.770 |  |  |
| ^a^ Regression coefficient of spatial error variable.  AIC: Akaike Information Criterion | | | |

| Table S6. Result of geographically weighted regression model among HIV self-testing kit purchasing population in 2019 in mainland China | | |
| --- | --- | --- |
| Variable | Spatial Correlation | City (coefficient)^a^ |
| Number of HIV testing facilities | Positive | (1) Sichuan Province: Luzhou (50.03), Yibin (48.10), Zigong (46.35), Neijiang (45.15), Ziyang (42.46), Leshan (41.06), Meishan (39.38), Suining (38.45), Guangan (37.32), Chengdu (35.59), Deyang (33.45)  (2) Guizhou Province: Bijie (46.98), Guiyang (44.60), Zunyi (44.42), Anshun (42.08), Liupanshui (39.82), Qiannan Buyi and Miao Autonomous Prefecture (39.08), Southwest Guizhou Autonomous Prefecture (36.31)  (3) Yunnan Province: Zhaotong (43.04)  (4) Guangxi Province: Hechi (34.01) |
| Number of HIV testing facilities | Negative | (1) Yunnan Province: Dai Autonomous Prefecture of Xishuangbanna (-23.47), Dehong Autonomous Prefecture (-20.01), Lincang (-18.68), Pudong (-18.08), Baoshan (-14.64), Dali Bai Autonomous Prefecture (-4.66)  (2) Tibet Province: Naqu (-19.98), Rikaze (-12.76), Lhasa (-8.07)  (3) Liaoning Province: Chaoyang (-8.67), Huludao (-7.25), Jinzhou (-6.34), Panjin (-4.65)  (4) Inner Mongolia Province: Chifeng (-7.94)  (5) Qinghai Province: Haixi Mongolian and Tibetan Autonomous Prefecture (-7.65)  (6) Xinjiang Province: Tarbagatay Prefecture (-6.56), Kelamayi (-6.56), Huyanghe (-5.80), Shihezi (-5.64), Wujiaqu (-4.96) |
| Urbanization ratio | Positive | (1) Sichuan Province: Luzhou (80.91), Yibin (70.91), Zigong (67.76), Neijiang (67.46), Ziyang (63.60),  Guangan (61.86), Suining (57.39)  (2) Guizhou Province: Bijie (74.23), Zunyi (73.26), Guiyang (71.10), Anshun (68.54), Liupanshui (65.20),  Southwest Guizhou Autonomous Prefecture (62.33), Qiannan Buyi and Miao Autonomous Prefecture (59.58)  (3) Fujian Province: Fuzhou (59.66), Ningde (59.78), Putian (58.18)  (4) Yunnan Province: Zhaotong (59.51)  (5) Zhejiang Province: Wenzhou (59.84), Taizhou (55.33) |
| Urbanization ratio | Negative | (1) Yunnan Province: Nujiang of the Lisu Autonomous Prefecture (-23.41), Diqing Tibetan Autonomous Prefecture (-22.9), Baoshan (-16.56), Dehong Autonomous Prefecture (-14.63), Dali Bai Autonomous Prefecture (-13.82), Lijiang (-11.12), Lincang (-10.01)  (2) Sichuan Province: Tibetan Autonomous Prefecture of Garzê (-22.78)  (3) Tibet Province: Changdu (-20.71), Linzhi (-13.84)  (4) Qinghai Province: Tibetan Autonomous Prefecture of Golog (-14.84), Yushu Tibetan Autonomous Prefecture (-12.91), Tibetan Autonomous Prefecture of Huangnan (-12.21)  (5) Inner Mongolia Province: Xilingol League (-13.96)  (6) Hebei Province: Chengde (-13.26), Tangshan (-13.22)  (7) Shandong Province: Dongying (-12.11), Weifang (-10.69)  (8) Tianjin (-11.55)  (9) Gansu Province: Gannan Tibetan Autonomous Prefecture (-10.55) |
| GDP | Positive | (1) Yunnan Province: Dehong Autonomous Prefecture (1.75), Baoshan (1.75), Nujiang of the Lisu Autonomous Prefecture (1.73), Lincang (1.72), Diqing Tibetan Autonomous Prefecture (1.69), Dali Bai Autonomous Prefecture (1.68), Puer (1.67), Dai Autonomous Prefecture of Xishuangbanna (1.66), Lijiang (1.61), Chuxiong (1.54)  (2) Tibet Province: Changdu (1.75), Linzhi (1.74)  (3) Qinghai Province: Yushu Tibetan Autonomous Prefecture (1.74), Tibetan Autonomous Prefecture of Golog (1.68), Tibetan Autonomous Prefecture of Huangnan (1.57), Xining (1.54), Tibetan Autonomous Prefecture of Haibei (1.53), Haidong (1.51)  (4) Sichuan Province: Tibetan Autonomous Prefecture of Garzê (1.64)  (5) Hainan Province: Tibetan Autonomous Prefecture of Hainan (1.63) |
| $R^{2}$ | 0.965 |  |
| AIC | 6152.120 |  |
| ^a^ Cities with top twenty for coefficient of spatial correlation  AIC: Akaike Information Criterion | | |
